# Antitumoral effects of attenuated *Listeria monocytogenes* in a genetically engineered mouse model of melanoma

**DOI:** 10.1038/s41388-019-0681-1

**Published:** 2019-01-21

**Authors:** Marianna Vitiello, Monica Evangelista, Nicole Di Lascio, Claudia Kusmic, Annamaria Massa, Francesca Orso, Samanta Sarti, Andrea Marranci, Katarzyna Rodzik, Lorenzo Germelli, Dinesh Chandra, Alessandra Salvetti, Angela Pucci, Daniela Taverna, Francesco Faita, Claudia Gravekamp, Laura Poliseno

**Affiliations:** 1 Institute of Clinical Physiology, CNR, Pisa, Italy; 2 Oncogenomics Unit, Core Research Laboratory, ISPRO, Pisa, Italy; 3 Molecular Biotechnology Center (MBC), University of Torino, Torino, Italy; 4 Department of Molecular Biotechnology and Health Sciences, University of Torino, Torino, Italy; 5 Center for Complex Systems in Molecular Biology and Medicine, University of Torino, Torino, Italy; 6 Department of Biochemistry and Molecular Biology, Centre of Postgraduate Medical Education, Warsaw, Poland; 7 Department of Microbiology and Immunology, Albert Einstein College of Medicine, New York, USA; 8 Unit of Experimental Biology and Genetics, Department of Clinical and Experimental Medicine, University of Pisa, Pisa, Italy; 9 Histopathology Department, Pisa University Hospital, Pisa, Italy

**Keywords:** Cell biology, Melanoma, Experimental organisms, Biologics, Cell biology

## Abstract

Attenuated *Listeria monocytogenes* (Lm^at^-LLO) represents a valuable anticancer vaccine and drug delivery platform. Here we show that in vitro Lm^at^-LLO causes ROS production and, in turn, apoptotic killing of a wide variety of melanoma cells, irrespectively of their stage, mutational status, sensitivity to BRAF inhibitors or degree of stemness. We also show that, when administered in the therapeutic setting to Braf/Pten genetically engineered mice, Lm^at^-LLO causes a strong decrease in the size and volume of primary melanoma tumors, as well as a reduction of the metastatic burden. At the molecular level, we confirm that the anti-melanoma activity exerted in vivo by Lm^at^-LLO depends also on its ability to potentiate the immune response of the organism against the infected tumor. Our data pave the way to the preclinical testing of listeria-based immunotherapeutic strategies against metastatic melanoma, using a genetically engineered mouse rather than xenograft models.

## Introduction

Melanoma is the most aggressive form of skin cancer and remains a therapeutic challenge, especially when it reaches the metastatic stage [1]. Since one of the reasons behind aggressiveness resides in the marked ability displayed by melanoma cells to evade immune recognition, over the years many different therapeutic strategies have aimed at potentiating the immune response of the organism against the tumor. In particular, antibodies that unleash the full potential of helper and killer T-lymphocytes have already become a standard of care for metastatic melanoma patients [2], while the enhanced recognition of cancer neo-antigens holds great promise [3–5].

Spurred by these successful applications of systemic immunotherapy, we have decided to assess the antitumoral effects elicited by an attenuated and non-pathogenic version of *Listeria monocytogenes* (Lm^at^). Among the microorganisms exploited as anticancer vaccines, Lm^at^ was chosen in light of the strong and multifaceted immune response that it triggers [6, 7], of its selective tropism for cancer cells [8], as well as of its tractability and versatility as drug delivery platform [9].

Here we show that the Lm^at^-LLO strain [9] very efficiently kills a broad spectrum of melanoma cells in culture and, when injected in the therapeutic setting in a genetically engineered mouse model (GEMM) of melanoma [10], it greatly impairs the growth and metastatic burden of melanoma tumors.

## Results

**Fig. 1:**
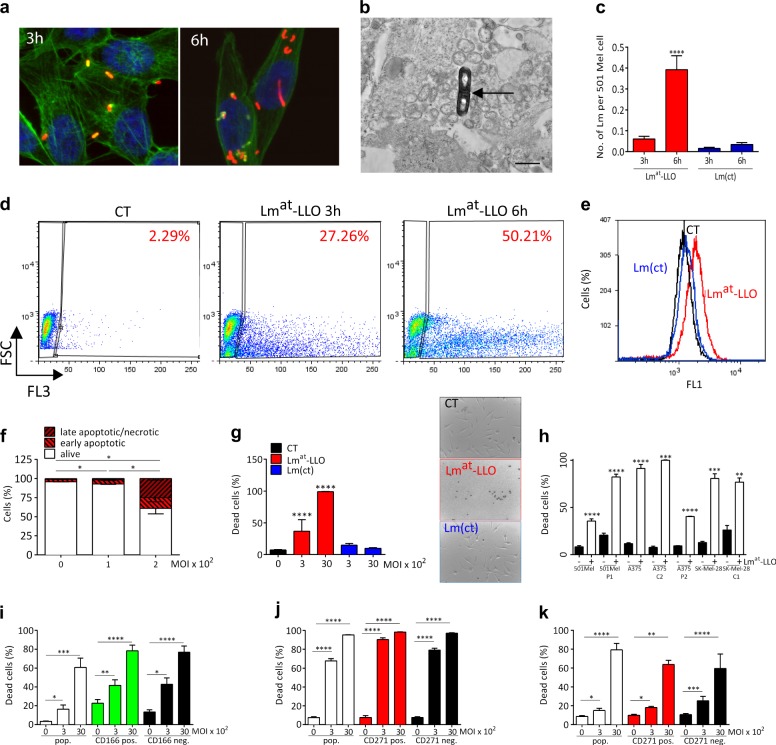
Lm^at^-LLO infects and kills melanoma cells. **a–c** Lm^at^-LLO is able to replicate inside melanoma cells, as determined using immunofluorescence (**a**), electron microscopy (**b**), and infection rate (**c**). In **a** 501 Mel cells were infected with MOI 3000 of Lm^at^-LLO for 3 h (*left panel*) or 6 h (*right panel*). Lm^at^-LLO is stained in red using anti-Listeria antibody, the F-actin of tumour cells is stained in green using fluorescent Phallotoxin and the nuclei are stained in blue using DAPI. The red clusters indicate that Lm^at^-LLO is able to replicate in the cytoplasm of melanoma cells. Original magnification: 63×. In **b** an example of listeria in division is captured using transmission electron microscopy. The black arrow indicates the septum that divides daughter cells (see Supplementary Fig. 2; scale bar: 500 nm). In **c** 501 Mel cells were infected with MOI 200 of Lm^at^-LLO. The increased infection rate when 3 h and 6 h are compared confirms that Lm^at^-LLO is able to replicate inside melanoma cells. Lm(ct), which lacks LLO expression, is used as negative control. **d** Lm^at^-LLO can spread across melanoma cells. 501 Mel cells were infected with MOI 200 of Lm^at^-LLO for 3 h or 6 h. Intracellular Lm^at^-LLO levels were evaluated using the anti-Listeria antibody. The increasing number of fluorescent cells indicates that Lm^at^-LLO is able to spread from cell to cell. Uninfected 501 Mel cells are used as control (CT). **e** Infection of 501 Mel cells with Lm^at^-LLO but not with Lm(ct) (MOI 200 for 6 h) causes ROS production. **f** As a consequence of ROS production, an increase in AnnexinV-positive apoptotic cells is observed. **g** Kill rate of Lm^at^-LLO in 501 Mel cells. Alive and dead cells were counted by trypan blue staining after 24 h of exposure to Lm^at^-LLO or Lm(ct) (MOI 300 and 3000). Representative pictures of CT, Lm^at^-LLO MOI 3000 and Lm(ct) MOI 3000 are reported in the panels on the right. **h** Lm^at^-LLO is effective at killing melanoma cells that show acquired resistance to vemurafenib. Kill rate in parental 501 Mel, A375 and SK-Mel-28 melanoma cells, as well as in their vemurafenib-resistant derivatives are reported. 501 Mel P1 resistant population and A375 C2 resistant clone are characterized by the expression of BRAFV600E Δ[3-10] splicing variant; in the A375 P2 resistant population, there is the KRAS K117N mutation; SK-Mel-28 C1 resistant clone is characterized by the overexpression of *EGFR* and *PDGFRbeta* [12]. Alive and dead cells were counted by trypan blue staining after 24 h of exposure to Lm^at^-LLO at MOI 3000. **i–k** Lm^at^-LLO is effective at killing melanoma cells with different degree of stemness. **i** Kill rate on unsorted (pop), CD166 pos. and CD166 neg. SK-Mel-5 cells. **j** Kill rate on unsorted (pop), CD271 pos. and CD271 neg. SK-Mel-2 cells. **k** Kill rate on unsorted (pop), CD271 pos. and CD271 neg. SK-Mel-28 cells. Alive and dead cells were counted by trypan blue staining after 24 h of exposure to Lm^at^-LLO at MOI 3000. The graphs represent the mean ± SEM of three independent experiments. *p < 0.05, **p < 0.01, ***p < 0.001, ****p < 0.0001

The ability of Lm^at^-LLO [9] (for a description of listeria strains used in this article, please refer to Supplementary Fig. 1) to enter and replicate inside melanoma cells in culture was suggested by the immunofluorescent staining of intracellular clusters of bacteria (Fig. 1a) and was confirmed by transmission electron microscopy, since we captured dividing bacteria (Fig. 1b, Supplementary Fig. 2). Consistently, we detected an increase in Lm^at^-LLO infection rate over time (Fig. 1c). We also detected an increase in the number of listeria-positive melanoma cells, which indicates that Lm^at^-LLO is capable of spreading from cell to cell (Fig. 1d, Supplementary Fig. 2e). We then investigated whether Lm^at^-LLO infection results in cell mortality. Treating infected cells with CellROX reagent, we found that listeria causes the production of intracellular reactive oxygen species (ROS, Fig. 1e) [11]. This indeed results in apoptotic cell death (Fig. 1f, Supplementary Fig. 3), hence in a dramatic decrease in cell viability (Fig. 1g). Critically, none of the above-mentioned biological effects (replication inside melanoma cells (Fig. 1c), spreading across cells (Supplementary Fig. 4), ROS production (Fig. 1e) and cell killing (Fig. 1g)) was observed when we used the Lm(ct) strain, which is impaired in the expression of the bacterial protein LLO (Supplementary Fig. 1c). The different behavior displayed by Lm^at^-LLO and Lm(ct) attests that the biological effects observed with Lm^at^-LLO are consequences of the bacterial life cycle rather than of a general toxicity phenomenon.

Next, we aimed at assessing whether the ability of Lm^at^-LLO to kill melanoma cells is broad-spectrum. Indeed, we obtained similar kill rates in cell lines of different stage (primary and metastatic) and of different BRAF, NRAS, and NF1 mutational status (Supplementary Fig. 5). Interestingly, we also found that Lm^at^-LLO is effective at killing melanoma cells that are particularly refractory to treatment, such as those that display acquired resistance to vemurafenib due to different molecular mechanisms (Fig. 1h) [12] or those that are characterized by a higher degree of stemness (CD166-positive [13] SK-Mel-5 cells (Fig. 1i), CD271-positive [14] SK-Mel-2 and SK-Mel-28 cells (Fig. 1j-k, Supplementary Fig. 6).

Prompted by the strong effects observed in vitro, we moved on to the assessment of the antimelanoma activity exerted by Lm^at^-LLO in vivo. To this end, we exploited the tissue-specific and inducible GEMM of metastatic melanoma developed by Dankort and colleagues [10] (Supplementary Fig. 7). In this model, upon skin painting with 4-hydroxitamoxifen (4-HT), Pten is loxed out (Supplementary Fig. 8a) and Braf acquires the V600E mutation, so that primary melanomas develop on site within few weeks (Supplementary Fig. 8b). These tumors are highly pigmented (i.e. the neoplastic cells are melanin rich), and heavily vascularized (Supplementary Fig. 8c-h). Furthermore, their growth is very fast—mice require euthanasia within 6–7 weeks after tumor induction—and metastasize rapidly to regional lymph nodes (LNs) and to the lungs (Supplementary Fig. 9-10).

In order to test the efficacy of Lm^at^-LLO in a therapeutic setting, we painted the back of 6 week-old mice with 4-HT and started Lm^at^-LLO injections 4 weeks later, when the primary tumor reached 50–100 mm^3^. One injection of high dose Lm^at^-LLO (10^7^ CFU) in the tumor area was followed by 14 intraperitoneal (IP) injections of a lower Lm^at^-LLO dose (10^5^ or 10^6^ CFU), one per day (Supplementary Fig. 11). The single intratumoral injection of a high dose of listeria favors the accumulation of the bacterium in the tumor microenvironment, while the serial injection of low doses stimulate the immune system to react against the bacterium itself [15]. The maximum dose of Lm^at^-LLO to be injected IP (10^6^ CFU) was chosen on the basis of IC50 calculation and absence of toxic side effects (Supplementary Fig. 12-13).

**Fig. 2:**
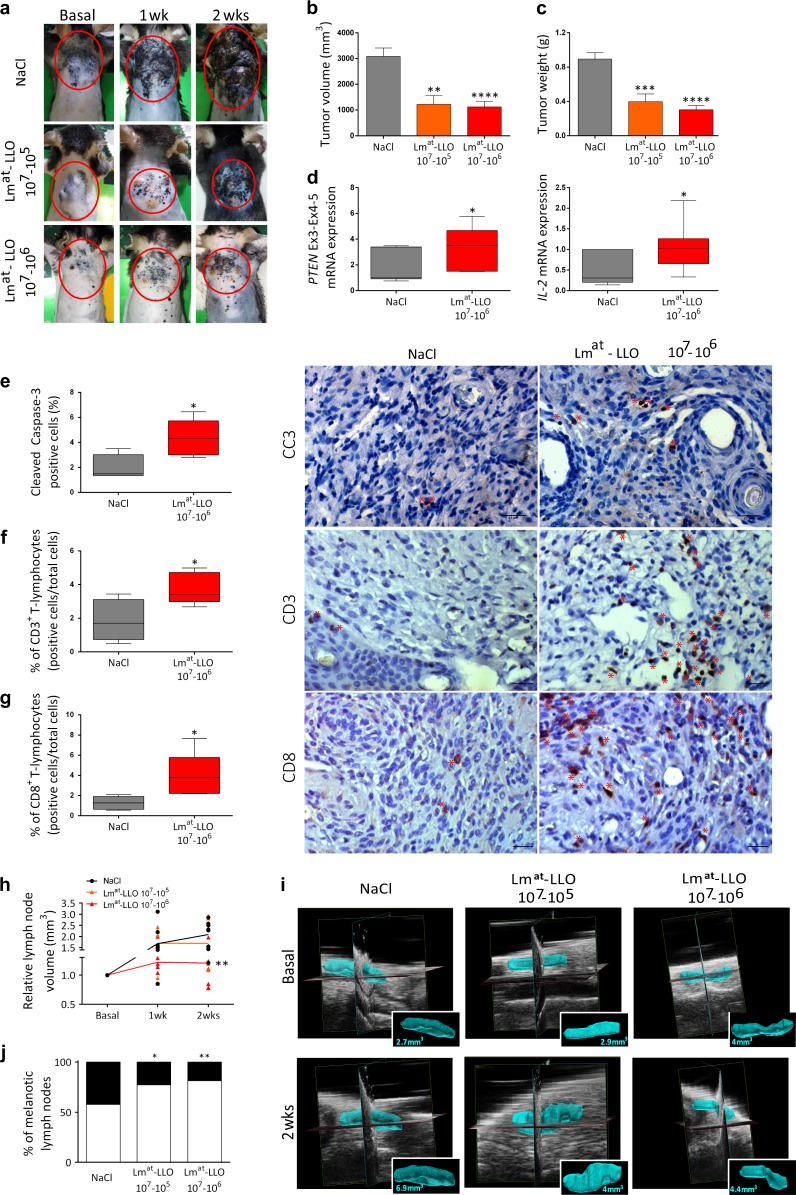
Lm^at^-LLO inhibits the growth of primary melanoma tumors and their metastatization to lymph nodes. **a–c** Lm^at^-LLO inhibits the growth of primary melanoma tumors. **a** Representative pictures of primary tumors (red line) developed by mice treated with saline solution (NaCl) or the indicated doses of Lm^at^-LLO (one injection of 10^7^ CFU in the tumor area was followed by one IP injection per day of 10^5^ or 10^6^ CFU for 14 days). For each experimental group, pictures were taken at three time points (before treatment, after 1 week of treatment and after 2 weeks of treatment). **b**, **c** Volume (**b**) and weight (**c**) of primary tumors. The number of primary tumors (mice) analyzed are: 12 (volume) and 13 (weight) for the NaCl group, 7 (volume) and 8 (weight) for the Lm^at^-LLO 10^7^–10^5^ group and 12 (volume and weight) for the Lm^at^-LLO 10^7^–10^6.^group. **d** Expression levels of undeleted *Pten* mRNA and *IL-2* mRNA. Total RNA extracted from paraffin embedded primary tumor samples was analyzed by qRT-PCR. Left: Undeleted *Pten* mRNA levels were measured using a forward primer located on exon 3 and a reverse located on exon 4–5, as reported in [26]. The higher levels of undeleted *Pten* mRNA detected in mice treated with Lm^at^-LLO compared to control mice are consistent with the smaller size of primary tumors. Right: The higher levels of *IL-2* mRNA detected in Lm^at^-LLO treated mice compared to control mice provide a molecular confirmation of the induction of the immune system by the vaccine. **e** Infection of tumor cells with Lm^at^-LLO causes a significant increase in apoptotic cell death, as indicated by Cleaved Caspase-3 immunostaining. The number of primary tumors (mice) analyzed is five for each experimental group. Original magnification: 40× (scale bar: 25 μm). **f**, **g** Infection of tumor cells with Lm^at^-LLO causes a significant increase in T-lymphocytes infiltration, as indicated by immunostaining of CD3+ (**f**) and CD8+ (**g**) cells. The number of primary tumors (mice) analyzed is five for each experimental group. Original magnification: 40× (scale bar: 20 μm). In **b–g** the mean ± SEM is reported. *p < 0.05, **p < 0.01, ***p < 0.001, ****p < 0.0001. **h, i** Lm^at^-LLO inhibits melanoma metastatization to lymph nodes. **h** Volume of inguinal lymph nodes in mice treated with saline solution (NaCl) or the indicated doses of Lm^at^-LLO, as measured by ultrasound imaging. The volume of right and left inguinal lymph nodes was measured at three time points (before treatment, after 1 week and after 2 weeks of treatment). The total number of lymph nodes (mice) analyzed was eight (four) for each experimental group. In the graph, individual measurements are reported, as well as a line connecting mean values. **p < 0.01. **i** 3D ultra high-frequency ultrasound reconstruction of a representative inguinal lymph node per experimental group (with surface rendering and volume measurement reported as picture in picture). The upper images were acquired before treatment, while the lower images were acquired after 2 weeks of treatment. **j** Melanin content of axillary, brachial, and inguinal lymph nodes in mice treated with saline solution (NaCl) or the indicated doses of Lm^at^-LLO. The total number of lymph nodes (mice) analyzed was the following: 59 (13) for the NaCl group, 44 (8) for the Lm^at^-LLO 10^7^–10^5^ group, 59 (12) for the Lm^at^-LLO 10^7^–10^6^ group. Black, high melanin content; white, no or low melanin content. *p < 0.05, **p < 0.01

Lm^at^-LLO treatment causes a conspicuous decrease in both the volume and the weight of primary tumors (Fig. 2a–c), so that the normal tissue, where the CRE-mediated deletion of Pten has not occurred, becomes more represented (Fig. 2d, **left**). Consistently with the results obtained in vitro, the impairment in tumor growth caused by Lm^at^-LLO is associated with an increase in the number of apoptotic Cleaved Caspase 3-positive cells (Fig. 2e). However, we also detected an increase in the levels of IL-2, a cytokine that promotes the differentiation of T cells (Fig. 2d, **right**) [16], as well as in the number of CD3+ T-lymphocytes recruited on site (Fig. 2f). Specifically, we detected a more pronounced infiltration of the CD4+ (Supplementary Fig. 14) and the CD8+ (Fig. 2g) subpopulations, which are responsible for cell-mediated and cytotoxic immunity against listeria, respectively [7, 15, 17]. These results confirm that in vivo Lm^at^-LLO potentiates the response of the immune system against the tumor and, therefore, that its antitumoral effects are at least in part non-cell autonomous [18].

We also assessed the ability of Lm^at^-LLO treatment to impair the metastatic burden. Indeed, the measurements obtained by ultrasound imaging indicate a decrease in the volume of regional LN (Fig. 2h, i and Supplementary Fig. 15), which, according to visual inspection, correlates with a decrease in melanin/melanoma deposits (Fig. 2j). Consistently, we observed a decrease in the number of metastatic nodules in the lungs (Supplementary Fig. 16).

Lm^at^-LLO treatment was not accompanied by overall toxicity—it didn’t cause weight loss or liver lesions (Supplementary Fig. 17)—and was characterized by selective accumulation of bacteria at the anatomic sites of tumor growth, confirming the renowned tropism of listeria for cancer cells (Supplementary Fig. 18).

## Discussion

The data we obtained in vitro attest the ability of Lm^at^-LLO to kill a wide variety of melanoma cells. Therefore, they suggest that Lm^at^-LLO treatment could overcome one of the most challenging features displayed by melanoma tumors, namely their high degree of heterogeneity [19].

Even more importantly, the data obtained in Braf/Pten mice reveal for the first time that the activity of Lm^at^-LLO is strong enough to be observed in spite of the fact that the melanoma tumors, which form in a GEMM, are much more heterogeneous than those that form in the xenograft models used thus far [20]. Therefore, they offer the rationale for testing new first-line treatment strategies that revolve around Lm^at^-LLO and for doing so in a preclinical setting that resembles human melanoma more closely, hence is endowed with higher translational relevance.

Considering its high versatility as a drug carrier, we suggest that Lm^at^-LLO could be engineered to express melanoma-associated antigens [21], so that the immune system of the body is boosted even further, or conjugated with a BRAF or a MEK inhibitor, providing an alternative way to assess the combined effects of immunotherapy and targeted therapy [22].

In light of the results we obtained in vitro, it should be considered that injections of Lm^at^-LLO might be effective also as a second-line treatment, on Braf/Pten mice that have become resistant to BRAF [23] or MEK [10] inhibitors.

Finally, Braf/Pten mice can be used to evaluate if the effects of Lm^at^-LLO are durable in time and if the tumors that eventually start growing again remain sensitive to a re-challenge [20]. Moreover, if mice with the *Tyr::CreER*+, *BrafCA/*+, *Pten*+*/lox* genotype instead of the *Tyr::CreER*+, *BrafCA/*+, *Ptenlox/lox* genotype are used for the induction, primary tumors grow more slowly and morbidity becomes related to metastases [24]. Therefore, a more thorough assessment of the effects of Lm^at^-LLO on metastatic sites can be obtained.

## Materials and methods

### Strains of *Listeria monocytogenes*

The characteristics of the strains of *Listeria monocytogenes* used in this work (Lm^at^-LLO and Lm(ct)) are described in Supplementary Fig. 1.

To obtain Lm^at^-LLO, the XFL-7 strain was electroporated with pGG-34 plasmid as reported in [25].

Analogously, to obtain Lm(ct), the XFL-7 strain was electroporated with the pGG-34-OVA_214-386_ plasmid. Both strains were subsequently grown in BHI medium with 34 μg/ml chloramphenicol at 37 °C.

### Mice strain

B6.Cg-Braf^tm1Mmcm^ Pten^tm1Hwu^ Tg(Tyr-cre/ERT2)13Bos/BosJ mice were purchased from the Jackson Laboratory (013590). For in vivo experiments, Tyr::CreER+ (heterozygous for CreER), BrafCA/+ (heterozygous for BrafV600E), and Ptenlox/lox (homozygous for Pten loss) mice were used, as described in Supplementary Fig. 11. Mice were treated in compliance with the animal protocol #754/2015-PR, initially approved by the Italian Ministry of Heath on July 27th 2015 and then amended on September 12th 2018.

### Statistical analyses

Statistical analyses were performed using unpaired and two-tailed Student *t*-test or Chi-square test, as needed. Values of *p* < 0.05 were considered statistically significant (*p < 0.05, **p < 0.01, ***p < 0.001, ****p < 0.0001).

## Supplementary information

Supplementary Figures S1-18

Supplementary Methods

Supplementary Table S1
