# Supplementary material for: Antitumoral effects of attenuated Listeria monocytogenes in a genetically engineered mouse model of melanoma
Source: Oncogene. 2019 Jan 21;38(19):3756–62. doi: 10.1038/s41388-019-0681-1 (PMC6756113; doi:10.1038/s41388-019-0681-1)
Supplement: Supplementary file 1 — Supplementary Figures S1-18 [file 41388_2019_681_MOESM1_ESM.pdf]

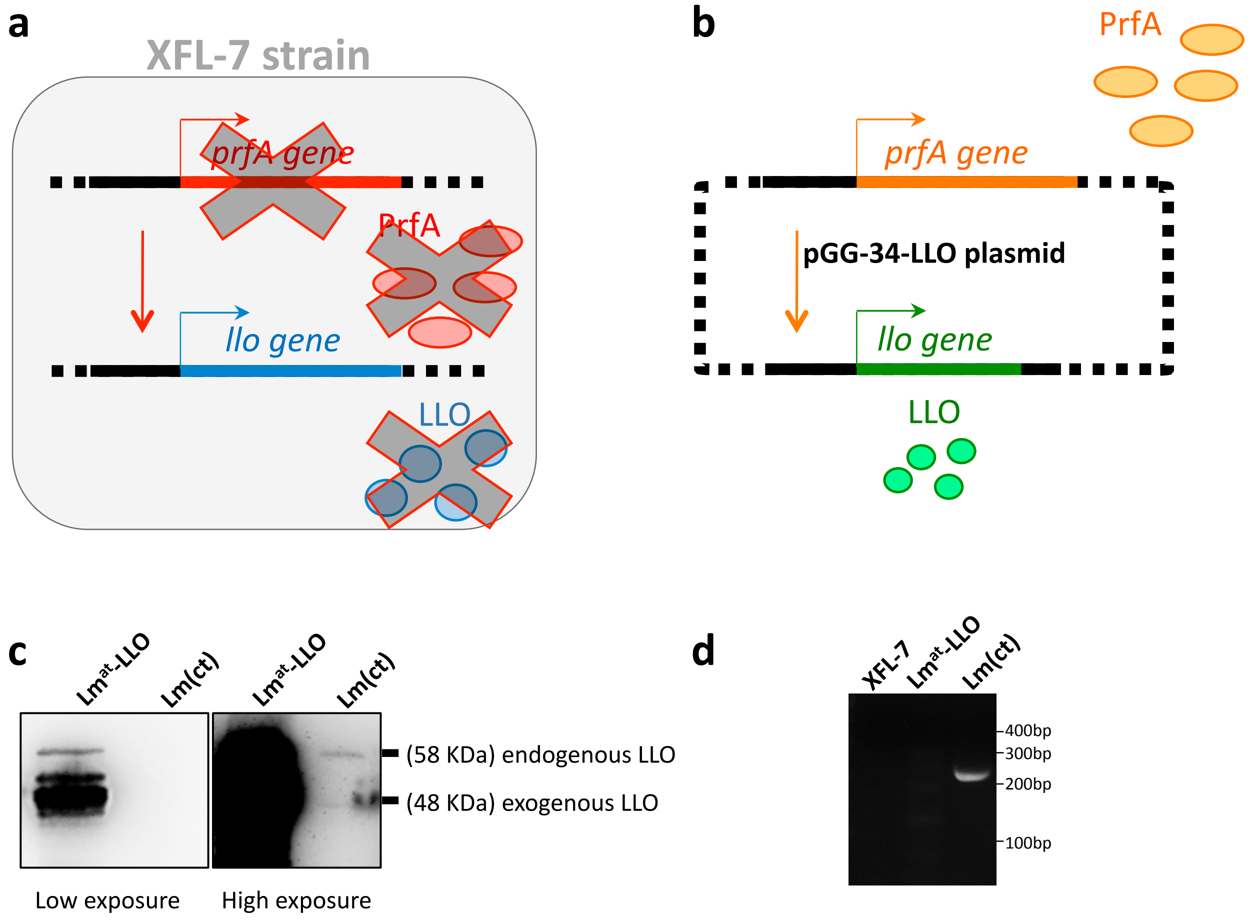

Supplementary Figure 1. The strains of *Listeria monocytogenes* used in this study.

Lm^{at}-LLO. The strain of this attenuated form of Listeria is XFL-7 **(a)**. The XFL-7 strain is characterized by the deletion of the *prfA* gene, which encodes for Positive Regulatory Factor A (PrfA), a transcription factor that is required by Listeria to regulate the expression of other virulent genes. Among them, Listeriolysin (LLO) is necessary to break free from cellular vacuoles and avoid digestion by lysosomes. When transformed with the pGG-34 plasmid, which drives the expression of both PfrA and LLO **(b)**, the XFL-7 strain re-acquires the ability to complete its life cycle. However, it shows a highly attenuated degree of virulence, because PrfA and LLO expressed from pGG-34-LLO contain multiple alterations. In particular, the LLO is truncated and, although still able to mediate the escape from the vacuole, its efficiency is reduced. Specifically, this is due to the fact that it lacks the C-terminal domain, which in turn is necessary for cholesterol binding and insertion into cell membranes. The western blot reported in **c** shows the endogenous (58KDa, upper band) and the exogenous (48KDa, lower band) LLO expressed by Lm^{at}-LLO.

Lm(ct). This strain is used as negative control for *in vitro* experiments. It was obtained from the XFL-7 strain by electroporation of the pGG-34-OVA$_{214\text{-}386}$ plasmid. This plasmid in turn drives the expression of a portion of chicken ovalbumin instead of LLO. The sequence of OVA can in fact be detected by PCR performed on genomic DNA **(d)**, while the expression of LLO is much lower than that observed in Lm^{at}-LLO and limited to the endogenous one **(c)**.

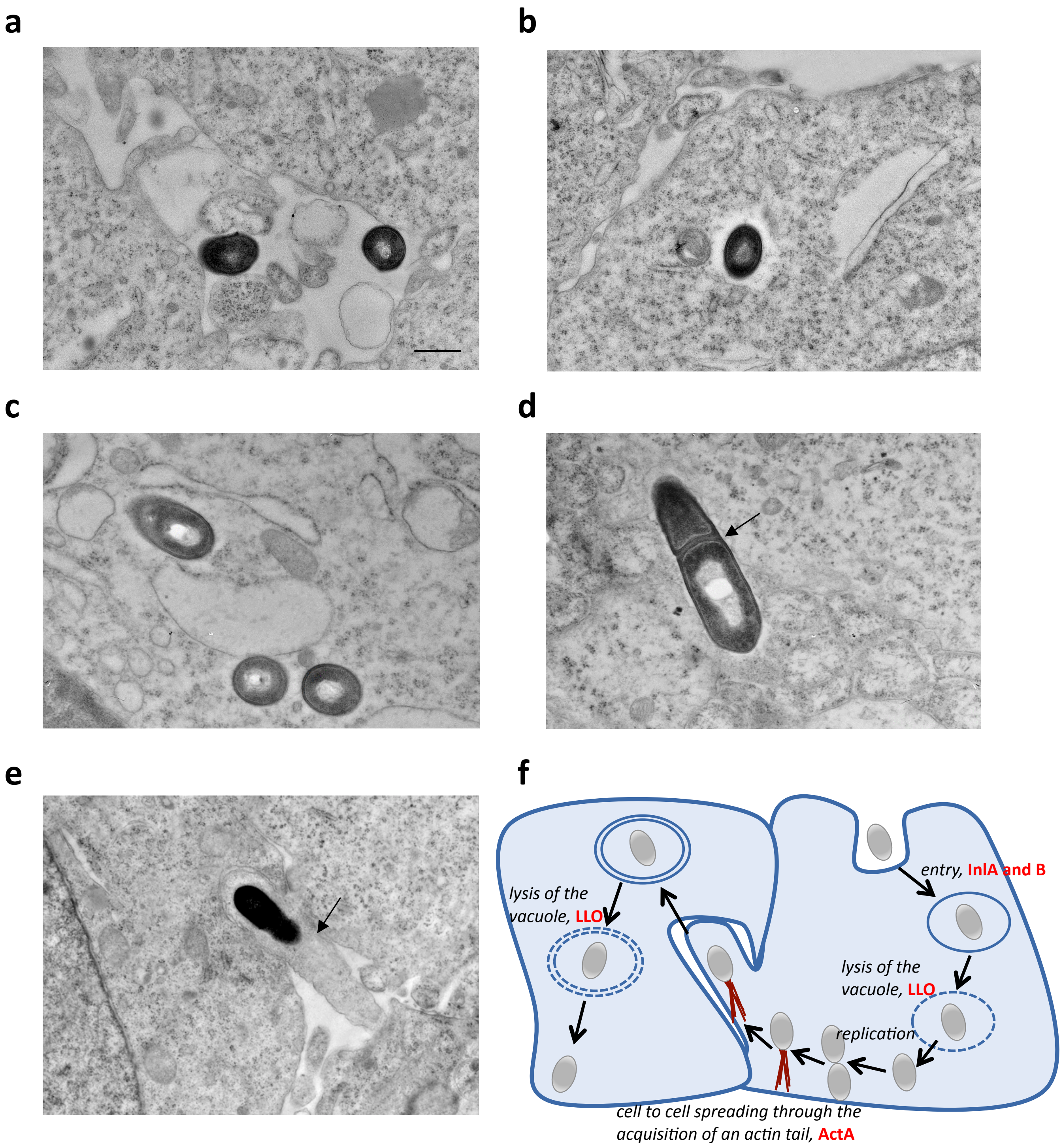

Supplementary Figure 2. Transmission electron microscope pictures of the Lm^{at}-LLO life cycle inside melanoma cells.

(a) Lm^{at}-LLO recognizes a cell of the 501 Mel line and mediates its own entry by Internalin A and B (InlA,B)-mediated fagocytosis. **(b)** Lm^{at}-LLO perforates the vacuole membrane by secreting LLO. **(c-d)** Lm^{at}-LLO replicates inside the cytoplasm of the host melanoma cell. The black arrow indicates the septum that divides daughter cells. **(e)** Thanks to ActA, Lm^{at}-LLO exploits actin polimerization for cell to cell spreading. The actin tail is well visible (black arrow). In **a-e** the pictures are representative of 3 independent experiments and the scale bar is 500nm. **(f)** The Lm^{at}-LLO life cycle is depicted in a cartoon.

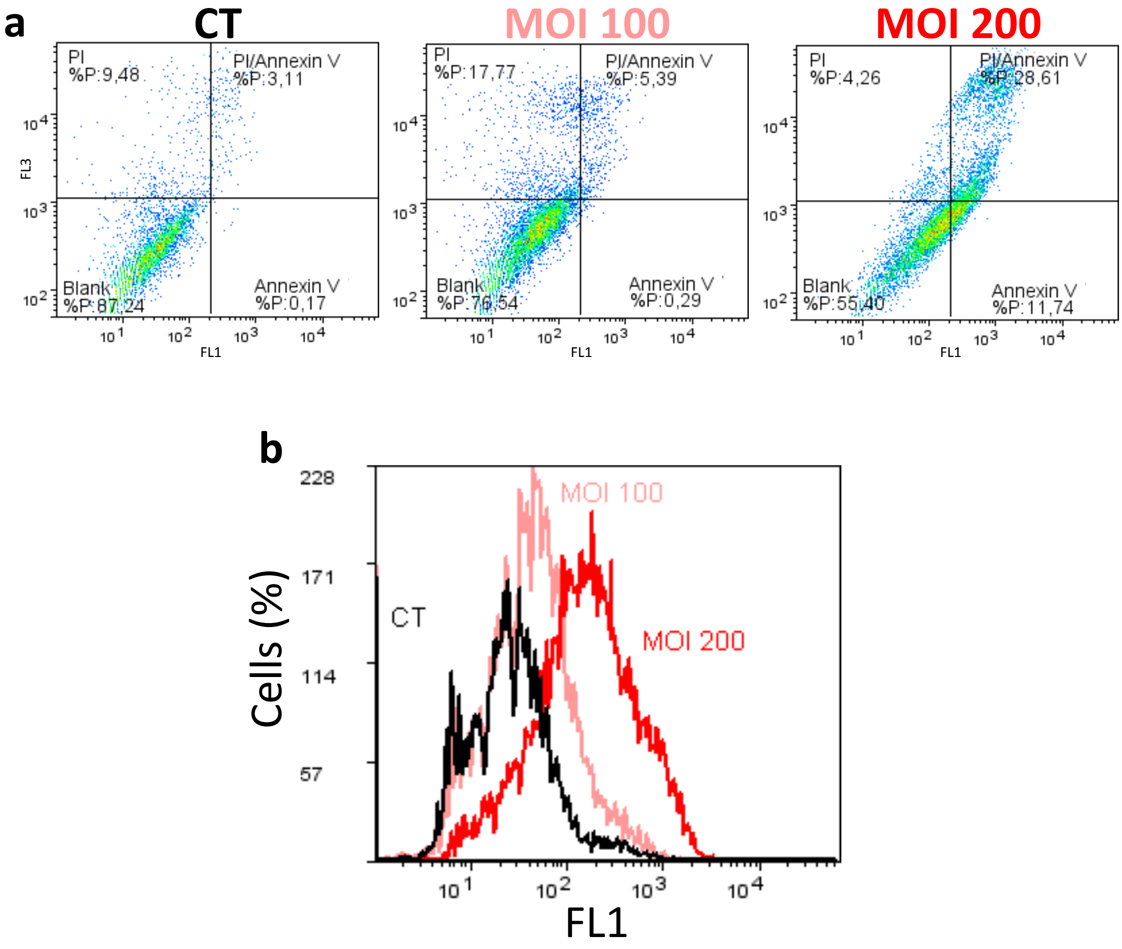

Supplementary Figure 3. Lm^{at}-LLO causes apoptotic cell death of melanoma cells in a dose-dependent manner.

(a) Representative FACS images of Annexin V/PI staining of 501 Mel cells exposed to the indicated MOI of Lm^{at}-LLO for 24h. **(b)** Representative percentages of Annexin V positive cells (upper and lower right quadrant in **a**). The quantification of early and late apoptotic cell percentages are reported in **Fig.1f**.

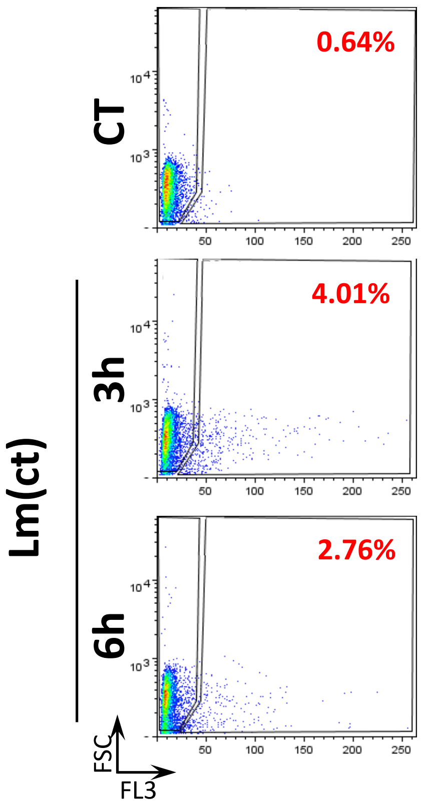

Supplementary Figure 4. Lm(ct) is not able to spread across melanoma cells.

501 Mel cells were infected with MOI 200 of Lm(ct) for 3h (*middle panel*) or 6h (*lower panel*) and intracellular levels of bacteria were evaluated using the anti-Listeria antibody. The fact that the percentage of fluorescent cells does not show an increase between 3 and 6h indicates that Lm(ct) is not able to spread from melanoma cell to melanoma cell. Uninfected 501 Mel cells are used as control (CT, *upper panel*). The pictures are representative of 3 independent experiments performed.

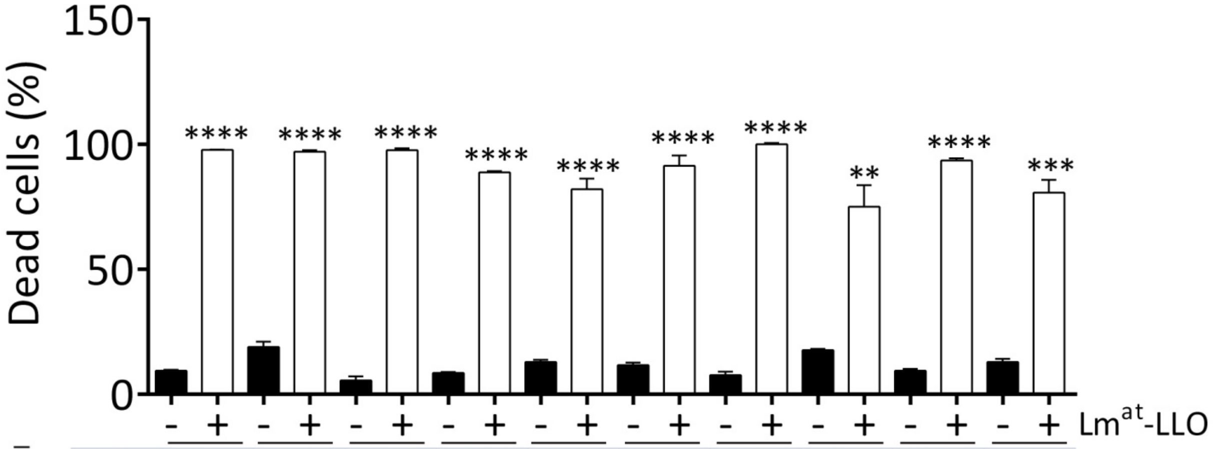

Mutational status										
cell line	WM35	WM278	WM793B	WM1361A	WM3211	501Mel	A375	SK-Mel-28	SK-Mel-2	MeWo
BRAF	**V600E**	**V600E**	**V600E**	wt	wt	**V600E**	**V600E**	**V600E**	wt	wt
NRAS	wt	wt	wt	**Q61R**	wt	wt	wt	wt	**Q61R**	wt
NF1	wt	wt*	wt	wt*	wt*	wt	wt*	wt*	wt*	**mut***
	mutBRAF			mutNRAS	triple wt	mutBRAF			mutNRAS	mutNF1
	Primary melanoma cell lines					Metastatic melanoma cell lines				

Supplementary Figure 5. Lm^{at}-LLO is able to kill all melanoma cells, irrespectively of their stage (primary or metastatic) and their mutational status (mutant BRAF, mutant RAS, mutant NF1, triple wt).

Alive and dead cells were counted by trypan blue to determine the kill rate of Lm^{at}-LLO administered at MOI 3000 for 24h. The graphs represent the mean±SEM of 3 independent experiments. **p<0.01, ***p<0.001, ****p<0.0001.

*reported in the literature. NF1 mutational status of WM278, WM1361A and WM3211 is reported in https://www.wistar.org/lab/meenhard-herlyn-dvm-dsc/page/melanoma-cell-lines-0. NF1 mutational status of A375 is reported in PMID 23288408 and PMID 27608486. NF1 mutational status of SK-Mel-2 and SK-Mel-28 is reported in PMID 27608486. NF1 mutational status of MeWo is reported in PMID 24576830 and PMID 27608486.

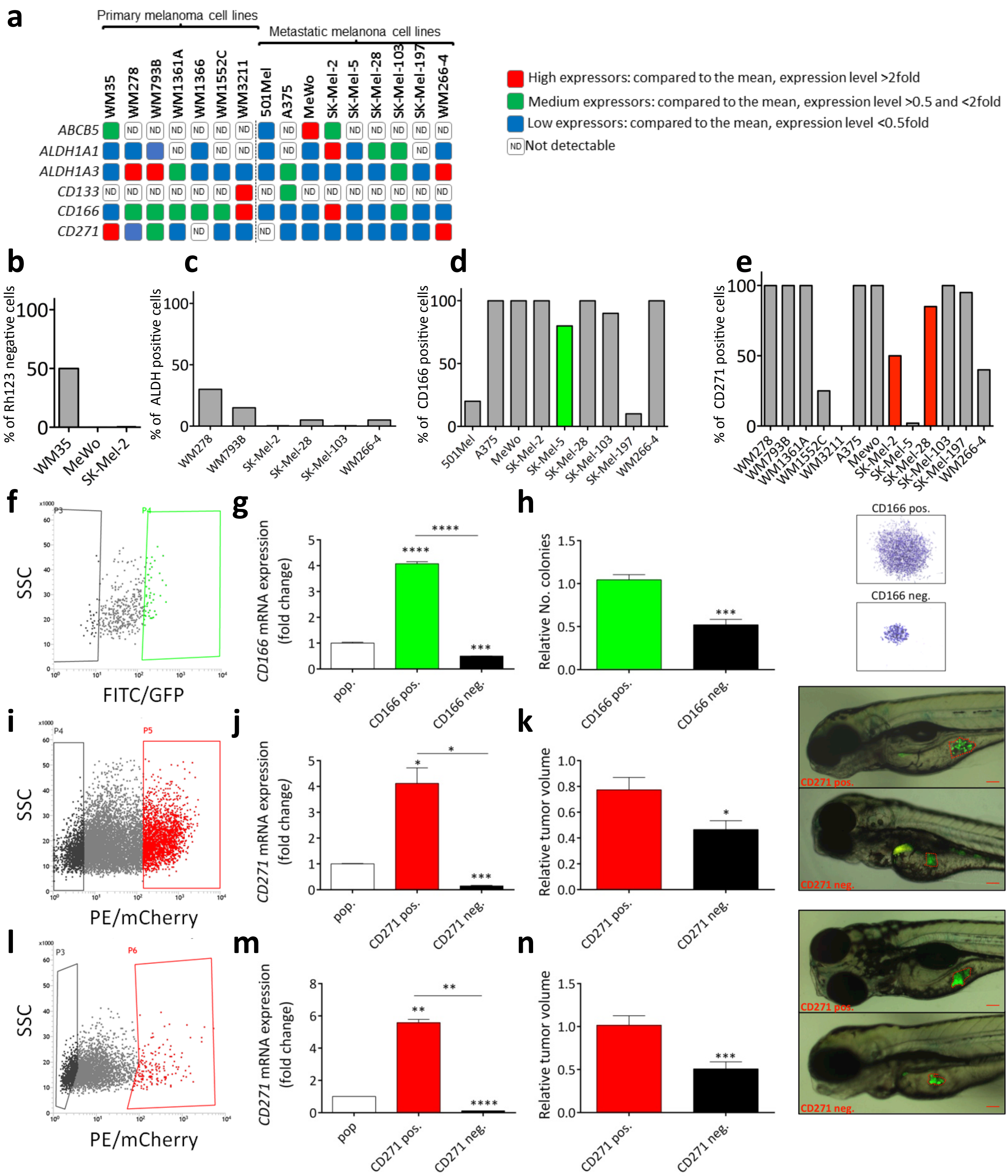

Supplementary Figure 6. Characterization of melanoma cells with different degree of stemness.

(a) qRT-PCR detection of the mRNA levels of stemness markers in melanoma cell lines. The cell lines to be tested in order to determine the corresponding protein levels were chosen among those that showed detectable levels of the corresponding mRNAs. **(b)** The number of cells that are positive for ABC transporters (including ABCB5) was determined in the indicated cell lines as percentage of cells that can release Rhodamine 123 (Rh123) and therefore are Rh123 negative. In spite of the high *ABCB5* mRNA expression levels, MeWo cells appear totally unable to release Rh123. **(c)** The number of cells that are positive for ALDH proteins was determined in the indicated cell lines as percentage of cells that can produce a fluorescent enzymatic compound using the Aldefluor^{TM} assay. **(d)** The number of CD166 positive cells was determined by FACS using an anti-CD166-VioBrightFITC antibody (Miltenyi). **(e)** The number of CD271 positive cells was determined by FACS using an anti-CD271-PE-Vio770 antibody (Miltenyi). **(f-h)** Analysis of CD166 positive and negative SK-Mel-5 cells. **(f)** SK-Mel-5 were sorted into CD166 positive and negative cells. **(g)** The differential *CD166* expression in the two sub-populations was confirmed by real-time PCR. **(h)** The ability to form colonies is higher in CD166 positive than in CD166 negative cells. **(i-k)** Analysis of CD271 positive and negative SK-Mel-2 cells. **(i)** SK-Mel-2 were sorted into CD271 positive and negative cells. **(j)** The differential *CD271* expression in the two sub-populations was confirmed by real-time PCR. **(k)** The ability to grow as xenografted tumor masses is higher in CD271 positive than in CD271 negative cells. After sorting, CD271 positive and negative SK-Mel-2 cells were injected into the yolk sac of 48hfp zebrafish embryos. The masses of the xenografted tumors were measured 48h later. Representative pictures of the tumors formed by CD271 positive and negative cells 48h post-injection are shown on the right. Scale bar: 100um. **(l-n)** Analysis of CD271 positive and negative SK-Mel-28 cells. **(l)** SK-Mel-28 were sorted into CD271 positive and negative cells. **(m)** The differential *CD271* expression in the two sub-populations was confirmed by real-time PCR. **(n)** The ability to grow as xenografted tumor masses is higher in CD271 positive than in CD271 negative cells. After sorting, CD271 positive and negative SK-Mel-28 cells were injected into the yolk sac of 48hfp zebrafish embryos. The masses of the xenografted tumors were measured 48h later. Representative pictures of the tumors formed by CD271 positive and negative cells 48h post-injection are shown on the right. Scale bar: 100um. The graphs represent the mean±SEM of 3 independent experiments. *$p<0.05$, **$p<0.01$, ***$p<0.001$, ****$p<0.0001$.

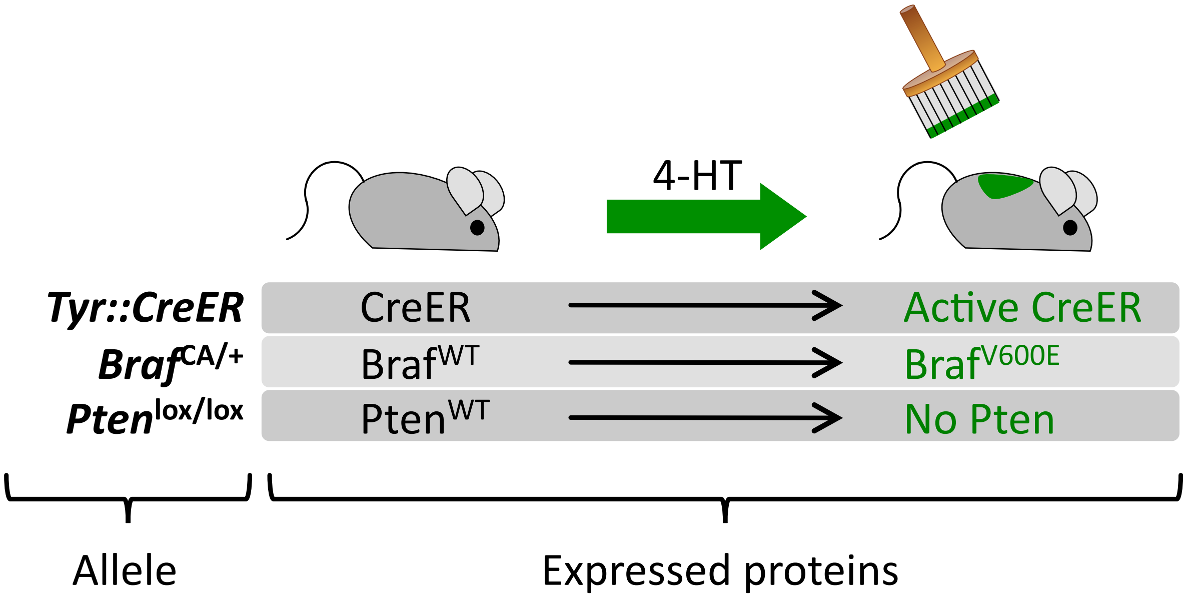

Supplementary Figure 7. The tissue-specific and inducible melanoma model used in this study.
B6.Cg-Braf^{tm1Mmcm} Pten^{tm1Hwu} Tg(Tyr-cre/ERT2)13Bos/BosJ mice were purchased from The Jackson Laboratory (#013590). In these mice, the expression of Cre enzyme is restricted to the melanocytic lineage because it is under the control of the promoter of Tyrosinase, an enzyme involved in melanin synthesis. Furthermore, Cre activity is inducible, because the protein is expressed as a fusion with the Estrogen Receptor (ER), which in turn mediates its translocation into the nucleus after skin painting with 4-hydroxitamoxifen (4-HT). Once in the nucleus, Cre causes the conversion of wt Braf into mutant BrafV600E and the loss of Pten (excision of exon 4 and 5), two hits that are sufficient for melanomagenesis.

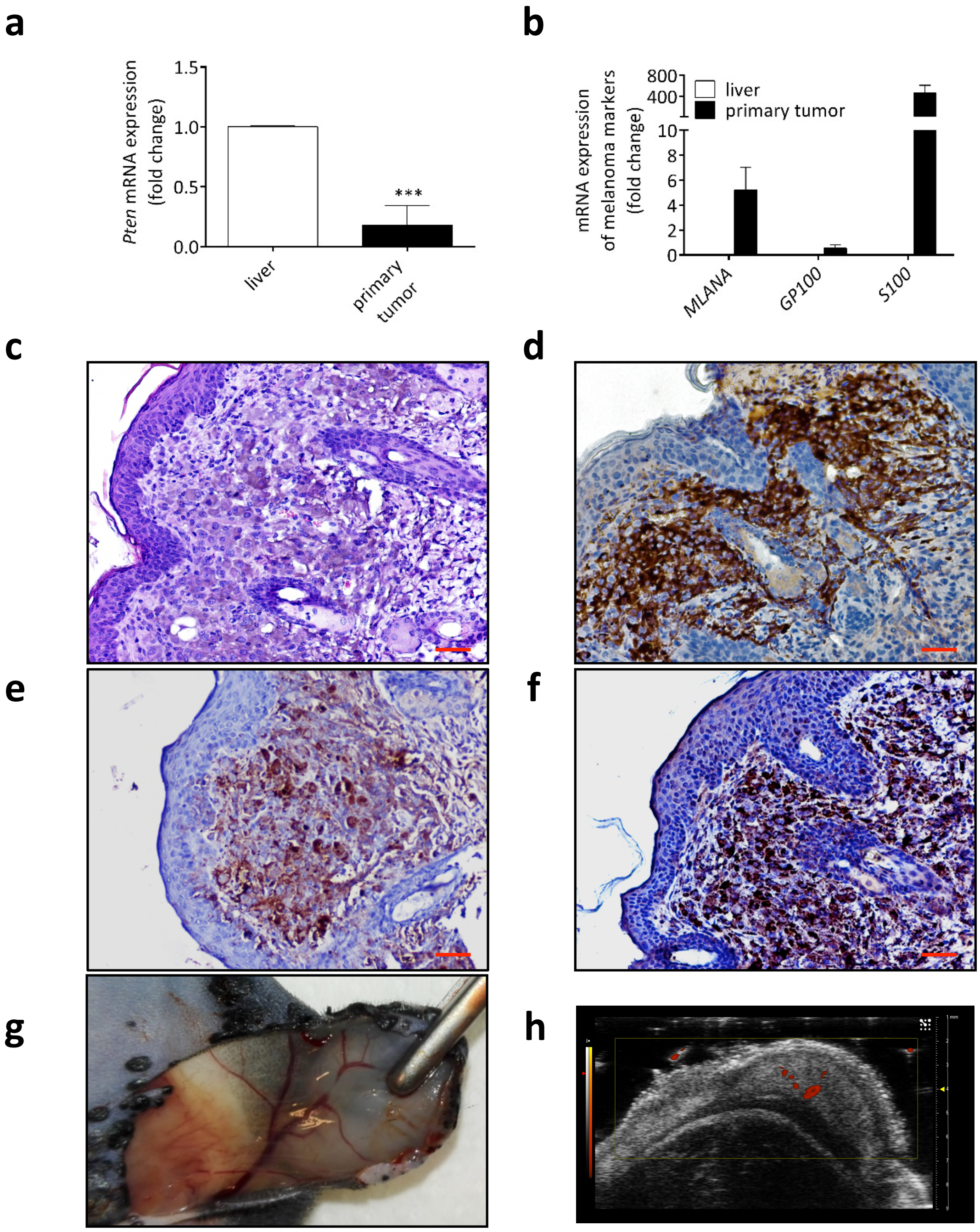

Supplementary Figure 8. Characterization of primary melanomas developed by *Tyr::CreER+,BrafCA/+,Ptenlox/lox* mice.

Within 4 weeks after painting of about 1cm^2 of shaved skin of the upper back with 4-hydroxitamoxifen (3ul of 5mM 4-HT, 3 days in a row), mice that are Tyr::CreER+ (heterozygous for CreER), BrafCA/+ (heterozygous for BrafV600E) and Ptenlox/lox (homozygous for Pten loss) develop primary melanomas around the site of painting. **(a-b)** Total RNA extracted from healthy liver and primary tumor was analyzed by qRT-PCR for the expression of Pten and melanoma markers MLANA, GP100 and S100. **(a)** *Pten* mRNA levels were detected using a forward primer located on exon 3 and a reverse primer located on exon 4-5 as reported in [Trotman et al., PLoS Biology 2003]. The lower levels detected in primary tumor compared to healthy liver are consistent with the floxing out of *Pten* exon 4-5 upon Cre induction by skin painting with 4-HT. **(b)** The higher levels of melanoma markers detected in primary tumor compared to healthy liver provide a molecular confirmation of the presence of tumor cells. Both in **a** and in **b** the indicated tissues were collected from 5 mice. Mean±SEM, ***p<0.001. **(c)** H&E staining of the primary tumor (original magnification: 20X; scale bar: 50um). **(d)** Combined Hematoxylin staining and melanin detection (original magnification: 20X; scale bar: 50um). **(e)** MLANA detection by immunoperoxidase staining (DAB chromogen substrate and Haematoxylin counterstaining. Original magnification: 20X; scale bar: 50um). **(f)** BRAFV600E detection by immunoperoxidase staining (DAB chromogen substrate and Haematoxylin counterstaining. Original magnification: 20X; scale bar: 50um). **(g)** Representative picture of a primary melanoma tumor that is highly vascularized. **(h)** B-mode ultra high-frequency ultrasound image of a primary melanoma tumor. The red patches represent blood vessels.

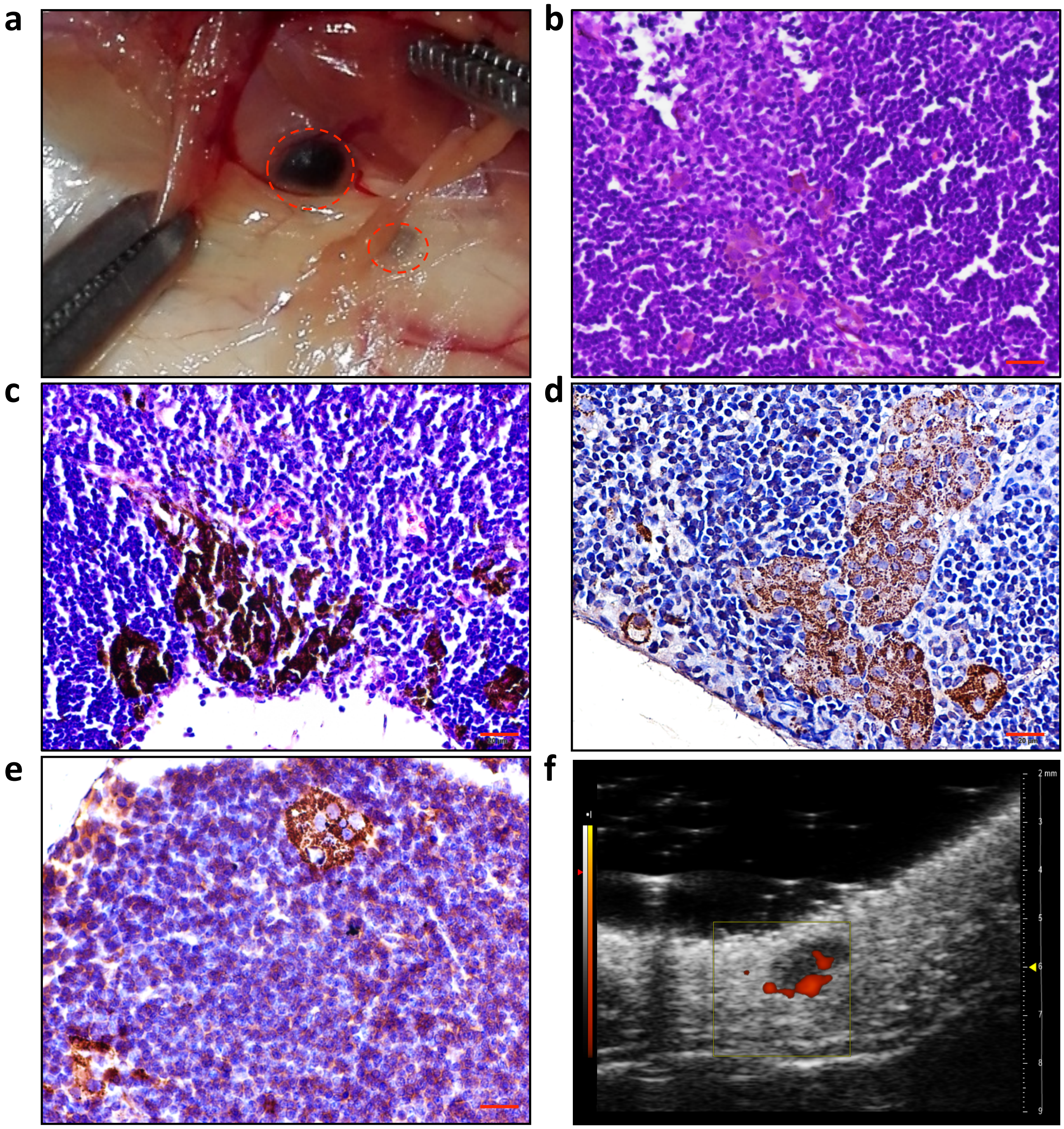

Supplementary Figure 9. Characterization of metastases to the regional lymph nodes (axillary, brachial and inguinal) developed by *Tyr::CreER+,BrafCA/+,Ptenlox/lox* mice.

Within 6 weeks after painting of *Tyr::CreER+,BrafCA/+,Ptenlox/lox* mice, melanoma cells reach the lymph nodes with 100% penetrance. **(a)** Representative picture of an axillary lymph node and a brachial lymph node that show melanin deposits. **(b)** H&E staining (original magnification: 40X; scale bar: 20um). **(c)** Combined Hematoxylin staining and melanin detection (original magnification: 40X; scale bar: 20um). **(d)** MLANA detection by immunoperoxidase staining (DAB chromogen substrate and Haematoxylin counterstaining. Original magnification: 40X; scale bar: 20um). **(e)** BRAFV600E detection by immunoperoxidase staining (DAB chromogen substrate and Haematoxylin counterstaining. Original magnification: 40X; scale bar: 20um). **(f)** B-mode ultra high-frequency ultrasound image of a inguinal lymph node. The red patches represent blood vessels.

The presence of MLANA and BRAFV600E-positive melanoma cells, together with the high degree of vascularization, indicate that regional lymph nodes are sites of metastatization.

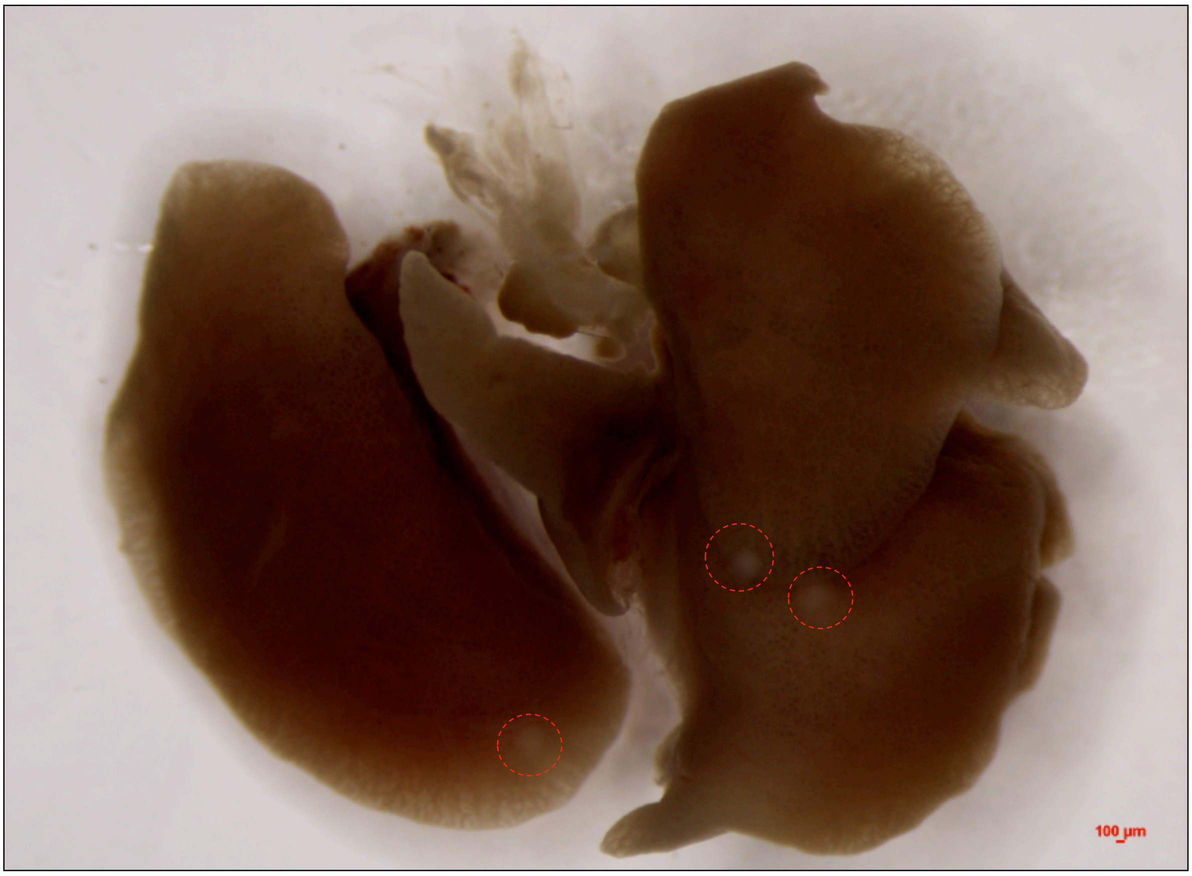

Supplementary Figure 10. Characterization of metastases to the lungs developed by *Tyr::CreER+,BrafCA/+,Ptenlox/lox* mice.

Within 6 weeks after painting of *Tyr::CreER+,BrafCA/+,Ptenlox/lox* mice, melanoma cells can be found in the lungs, as detected by visual inspection.

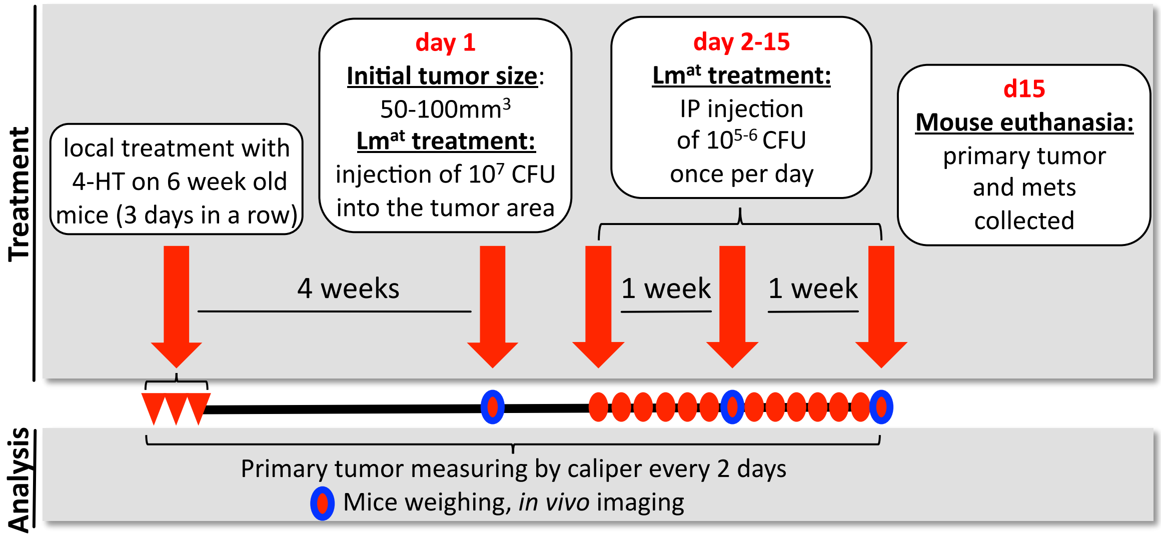

	In loco injections	IP injections
**Group 1**	50ul saline sol.	100ul saline sol.
**Group 2**	10^7 CFU/50ul	10^5 CFU/100ul
**Group 3**	10^7 CFU/50ul	10^6 CFU/100ul

Supplementary Figure 11. Description of the protocol used for the treatment of *Tyr::CreER+,BrafCA/+,Ptenlox/lox* mice with Lm^{at}-LLO.
Melanoma formation is induced in 6 week-old *Tyr::CreER+,BrafCA/+,Ptenlox/lox* mice (both males and females) by 4-HT treatment (3ul of 5mM 4-HT applied on about 1cm^2 of shaved skin of the upper back for 3 days in a row). 4 weeks later, when the primary tumor reaches a volume of 50-100mm^3, Lm^{at}-LLO injections are started. The injection protocol is as follows: on day 1, 10^7 CFU of Lm^{at}-LLO resuspended in 50ul of saline solution (NaCl) are injected in the tumor area; on day 2-15, 10^5 or 10^6 CFU of Lm^{at}-LLO resuspended in 100ul of saline (NaCl) solution are injected intraperitoneally (IP) once per day. The mock injections of saline solution are equivalent in volume.
On the first day of treatment with Lm^{at}-LLO, as well as after one and two weeks (last day of treatment), mice are weighted and the size of their primary tumor is measured by caliper. At the same time points, mice are also subjected to ultrasound imaging.

a

Lm^{at}-LLO CFU	# mice	Dead mice	Ratio dead/ total mice
NaCl	20	0	0
$5x10^8$	20	18	0.9
10^8	20	4	0.2
10^7	20	0	0

b

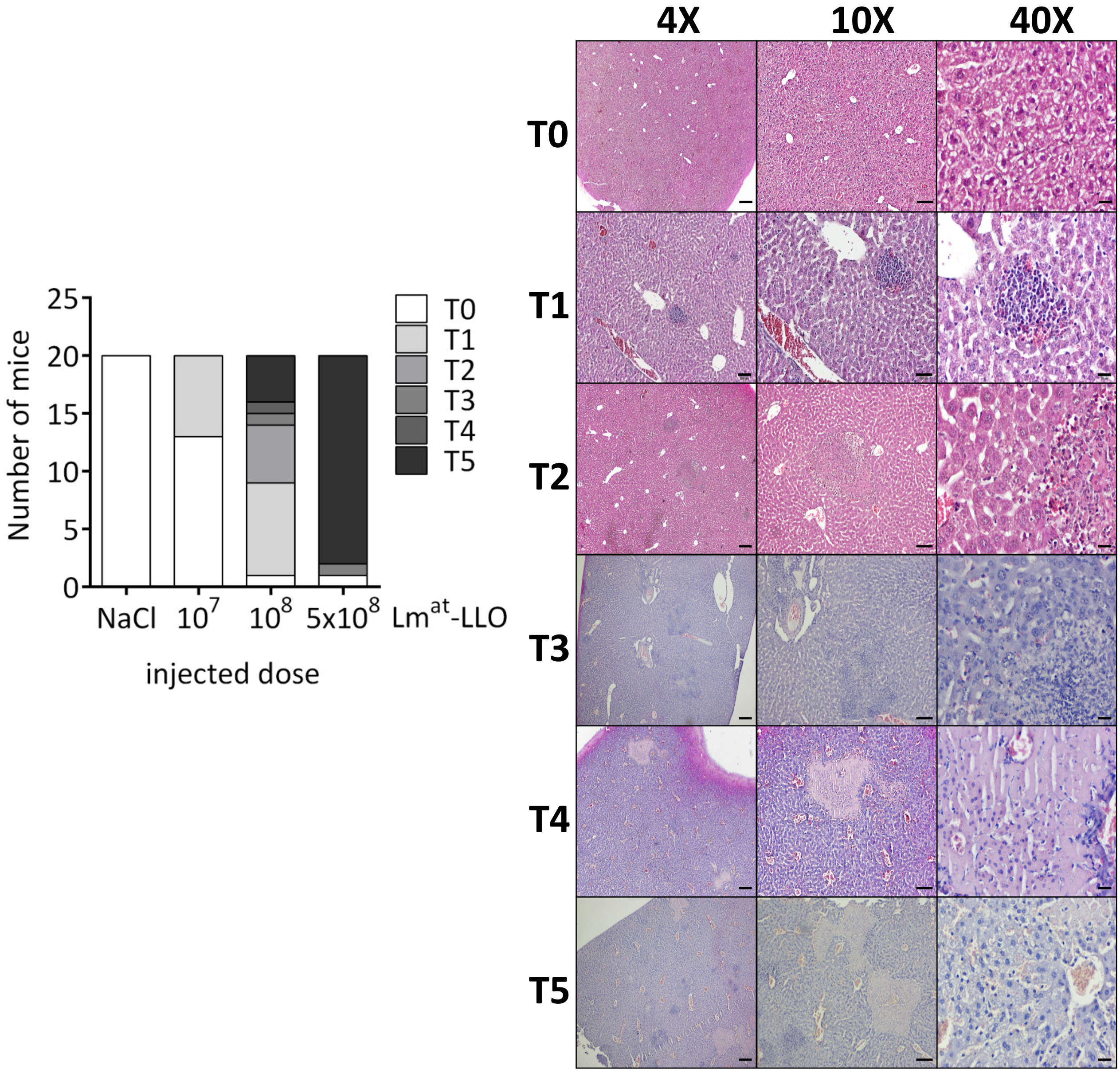

Supplementary Figure 12. LD50 of Lm^{at}-LLO.

The LD50 of Lm^{at}-LLO was determined by intraperitoneal injection of 20 mice with increasing doses of Listeria (10^7, 10^8 or $5x10^8$ CFU diluted in 100ul of saline solution) and subsequent monitoring for three days. **(a)** Percentage of dead mice over the three days period. The IC50 is reached between 10^8 and $5x10^8$ CFU. **(b)** At the end of the three days, the livers of the mice that were still alive were collected, analysed by H&E staining and classified as follows: T0 = no lesions; T1 = uniform light discolouration and firmness; T2 = white plaques visible covering 5% of the liver surface; T3 = white plaques covering 10% of liver surface; T4 = white plaques covering up to 30% of liver surface; T5 = white plaques covering more than the 30% of liver surface [Singh et al., BJC 2014]. Livers of dead mice were considered T5. The graph on the left summarizes the number of mice of each experimental group showing the indicated grade of toxicity, while a representative liver of each toxicity grade is shown on the right (H&E staining; original magnification: 4X (scale bar: 200um), 10X (scale bar: 100um) and 40X (scale bar: 20um)).

According to the LD50 calculation, in our experimental protocol we could use a systemic dose of Lm^{at}-LLO as high as 10^7 or even $2.5x10^7$ CFU. However, we decided to avoid liver toxicity at all. Therefore, we chose not to go higher than 10^6 CFU, also because we perform not just one, but repeated intraperitoneal injections.

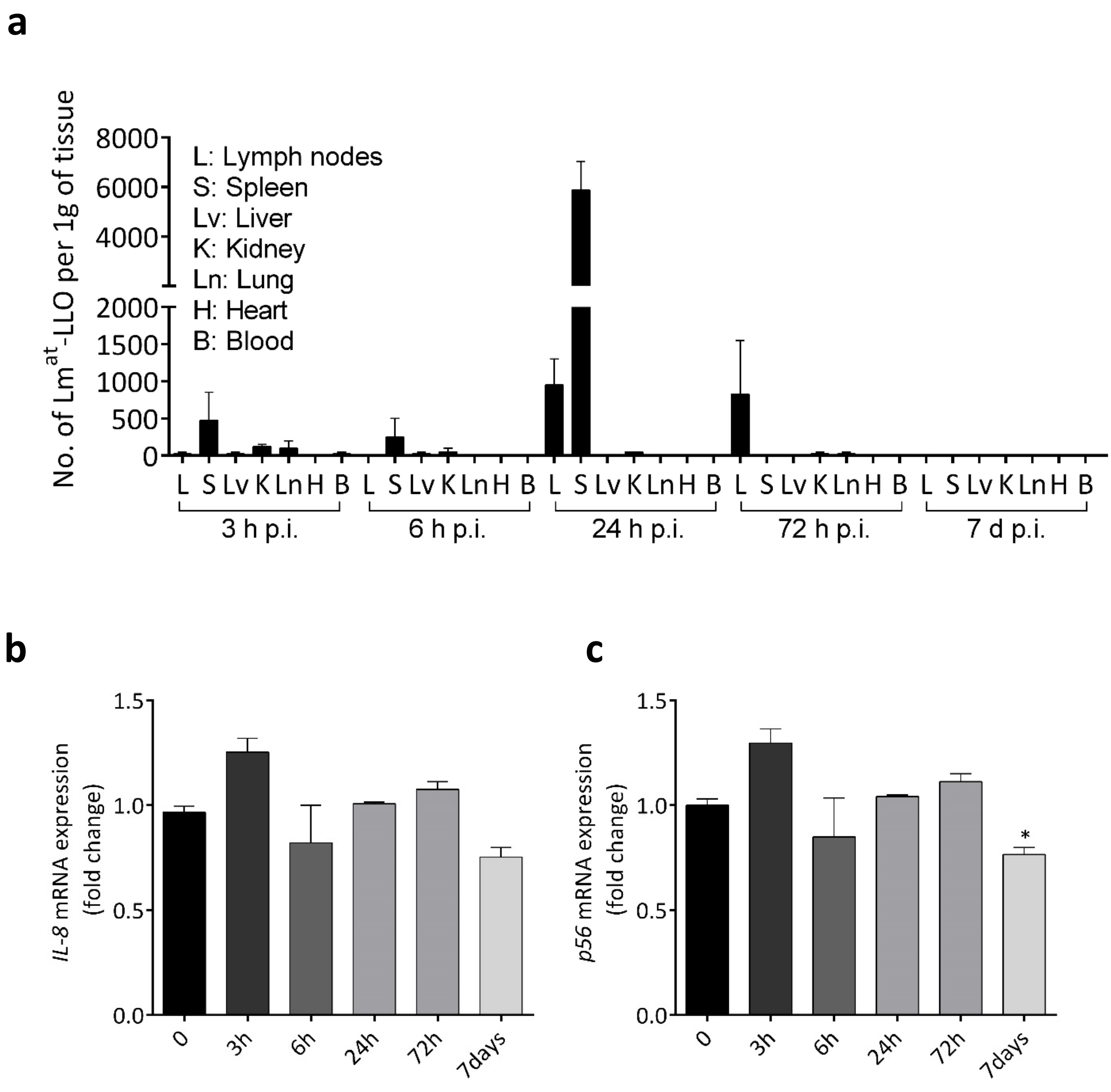

Supplementary Figure 13. IP injections of Lm^{at}-LLO at 10^6 CFU of do not affect healthy tissues.
(a) Biodistribution of Lm^{at}-LLO in different tissues at different time points (3h, 6h, 24h, 72h and 7 days) after intraperitoneal injection of 10^6 CFU of Lm^{at}-LLO in healthy mice. Listeria preferentially accumulates in lymph nodes and spleen but is completely cleared within 7 days. 3 mice were used for each time point and the mean±SEM is reported. **(b-c)** Healthy mice were injected IP with 10^6 CFU of Lm^{at}-LLO. Livers were collected at different time points after the injection (3h, 6h, 24h, 72h and 7 days) and the mRNA level of IL-8 **(b)** and p56 **(c)** were evaluated to test toxicity. 3 mice were used for each time point and the mean±SEM is reported. *$p<0.05$.

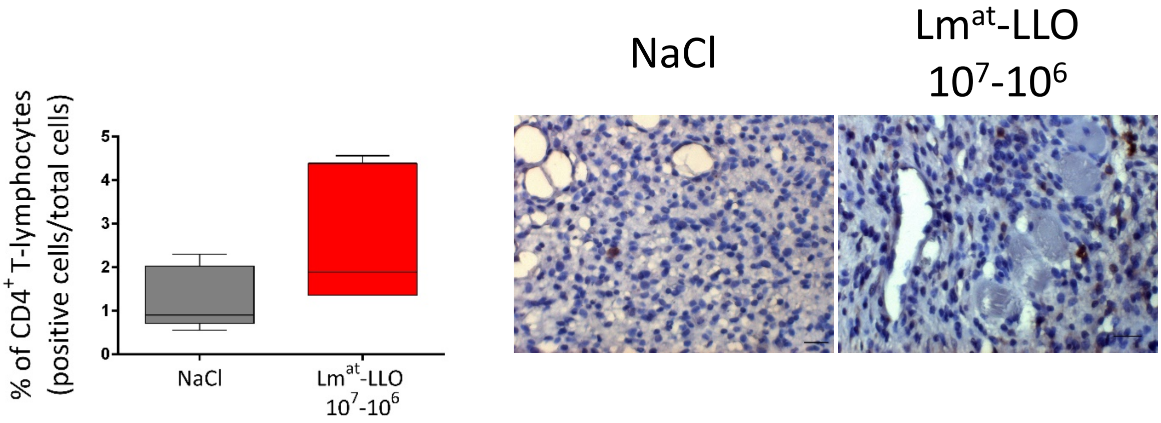

Supplementary Figure 14. CD4 positive T-lymphocytes.

Infection of tumor cells with Lm^{at}-LLO causes an increase (p-value = 0.0599) in the number of infiltrating CD4+ T-lymphocytes. The number of primary tumors (mice) is 5 for each experimental group. Original magnification: 40X (scale bar: 20um).

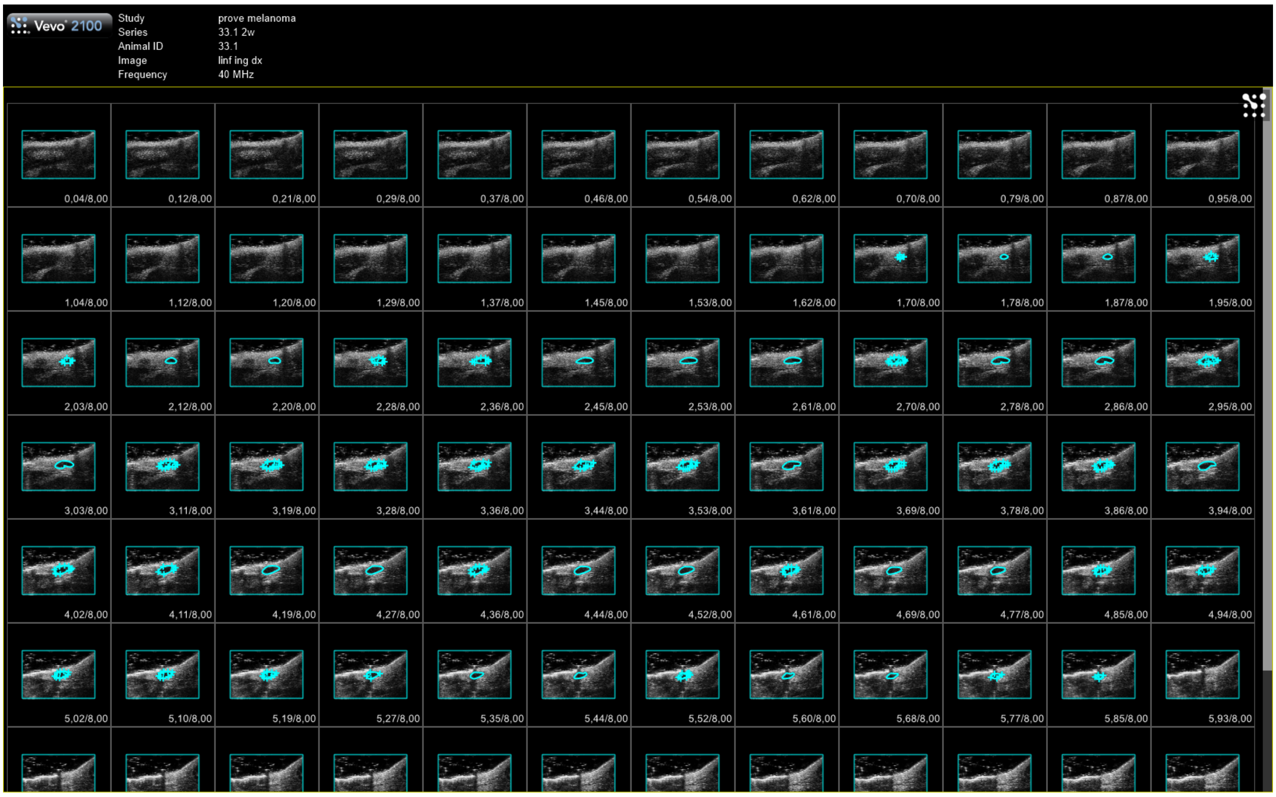

Supplementary Figure 15. B-mode ultra high-frequency ultrasound scans with semi automatic segmentation of lymph node area.
All images were acquired by a motorized probe in order to reconstruct 3D volumes.

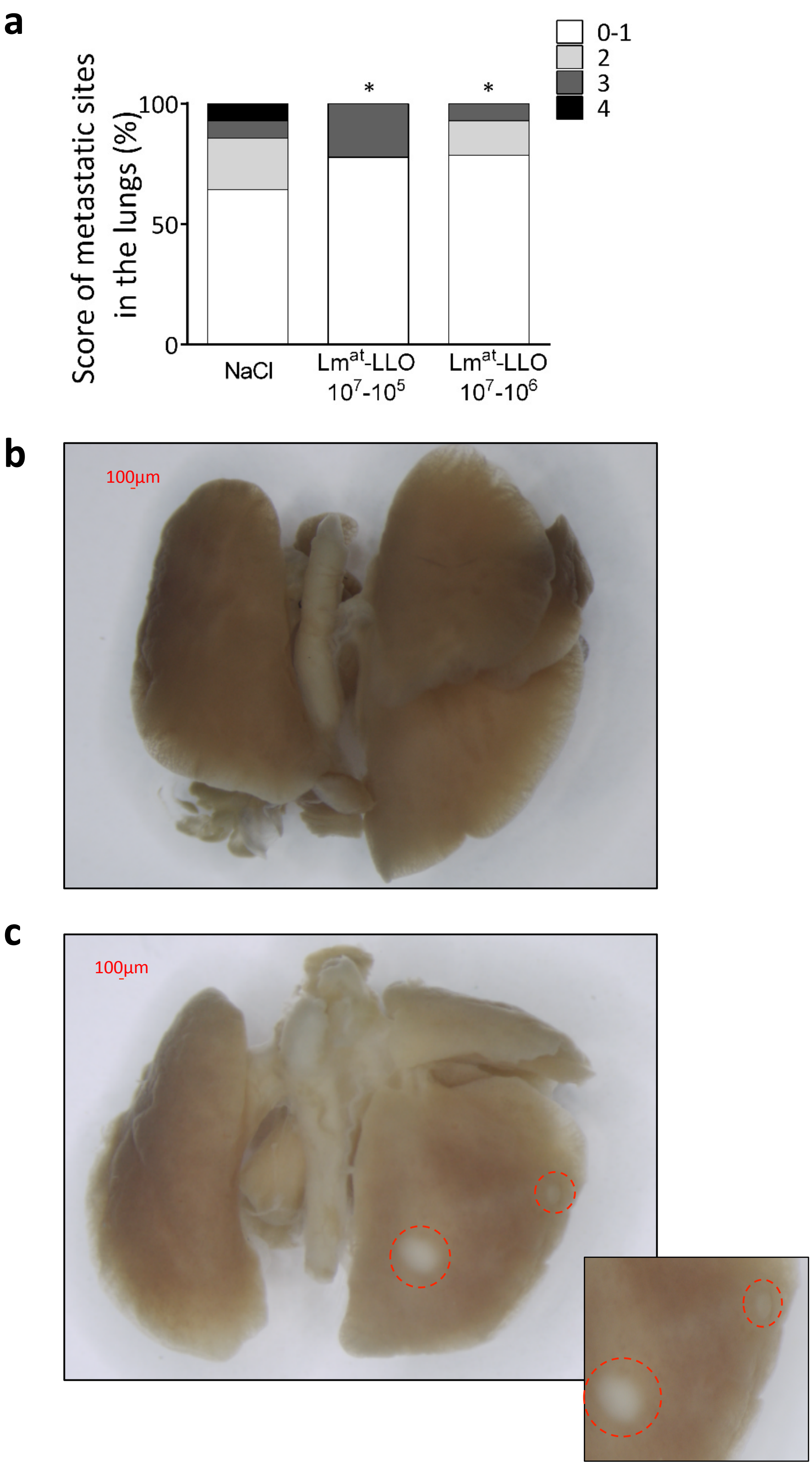

Supplementary Figure 16. Decrease in the number of lung metastasis upon treatment with Lm^{at}-LLO.
(a) Percentage of mice that developed the indicated number of lung metastases upon treatment with saline solution (NaCl) or Lm^{at}-LLO. *$p<0.05$. **(b-c)** Representative picture of a healthy **(b)** and a metastasized **(c)** lung. The enlarged image of the metastases is reported in the insert (dotted circles). Number of mice analyzed: 14 for the NaCl group; 9 for the Lm^{at}-LLO 10^7-10^5 group; 14 for the Lm^{at}-LLO 10^7-10^6 group.

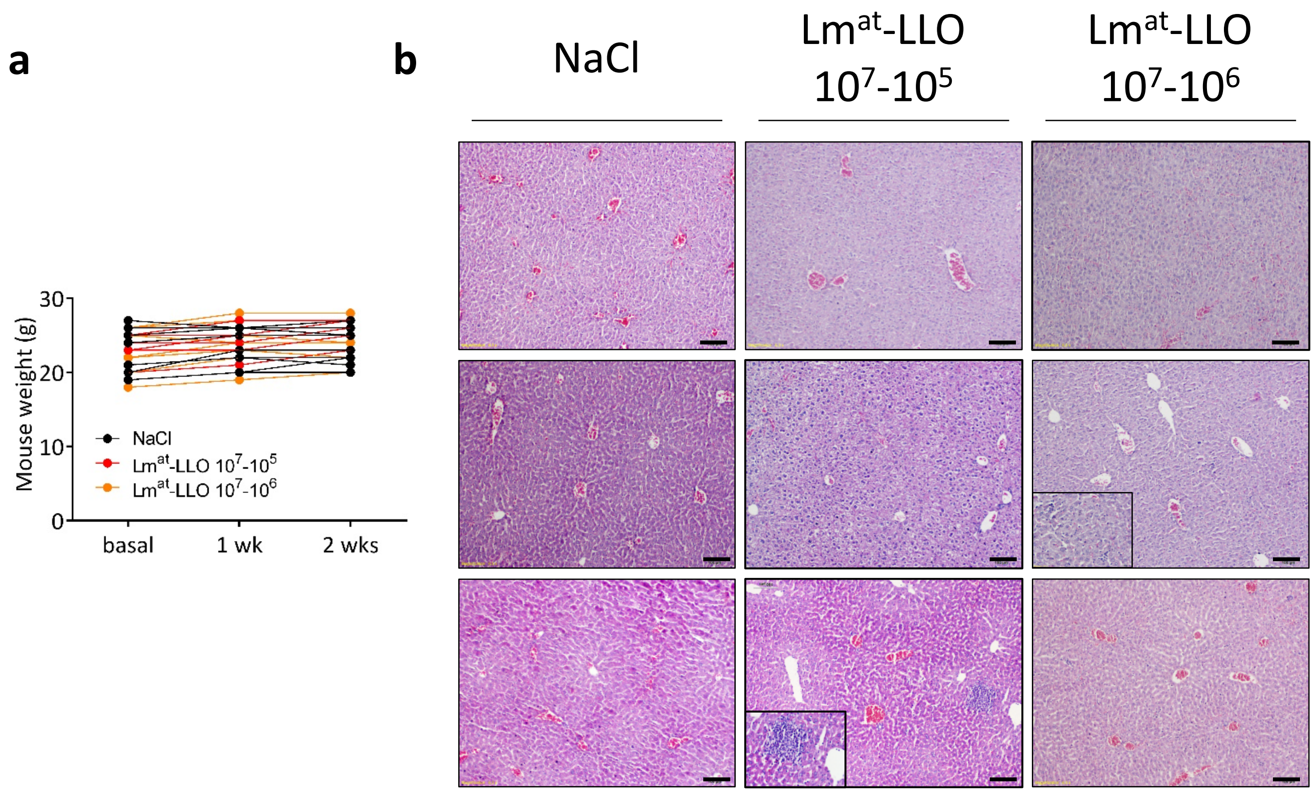

Supplementary Figure 17. Overall toxicity of treatment with Lm^{at}-LLO.
(a) Mice weight during the course of treatment. Number of mice analyzed: 13 for the NaCl group; 8 for the Lm^{at}-LLO 10^7-10^5 group; 11 for the Lm^{at}-LLO 10^7-10^6 group. **(b)** H&E staining of livers at the end of the treatment period in 3 representative mice per group. Livers of both control and treated mice can be classified as T0 (no lesions, see Supplementary Fig.12). Original magnification: 10X (scale bar: 100um). The absence of weight loss and liver toxicity indicates that listeria treatment is not accompanied by overall toxicity.

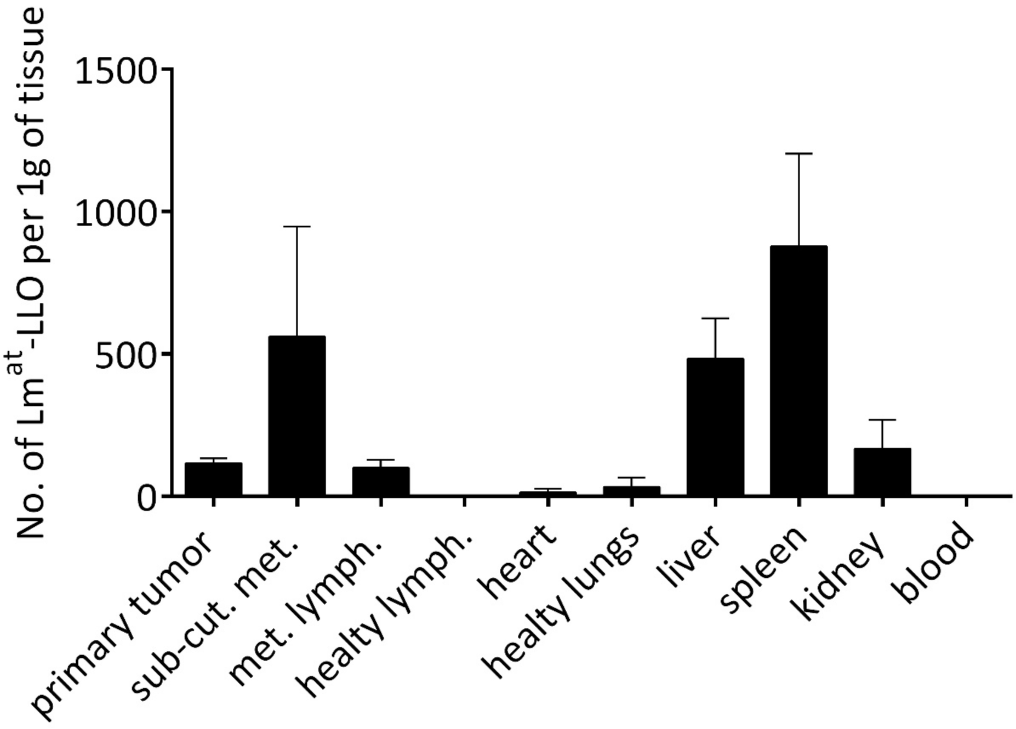

Supplementary Figure 18. Biodistribution of Lm^{at}-LLO in different tissues one day after the end of the experimental protocol (d16, no more than 24h after the last Lm^{at}-LLO injection).

Listeria accumulates preferentially in the primary tumor and in the organs where melanoma has metastasized, while tumor-free organs, such as healthy lymph nodes, heart, healthy lungs and blood, appear listeria-free.

The presence of listeria in the spleen, liver and kidney has to be considered physiological, because these organs are dedicated to blood filtering, as well as tissue detoxification and clearance.

Number of mice analyzed = 5.
