# Supplementary material for: Antitumoral effects of attenuated Listeria monocytogenes in a genetically engineered mouse model of melanoma
Source: Oncogene. 2019 Jan 21;38(19):3756–62. doi: 10.1038/s41388-019-0681-1 (PMC6756113; doi:10.1038/s41388-019-0681-1)
Supplement: Supplementary file 2 — Supplementary Methods [file 41388_2019_681_MOESM2_ESM.pdf]

SUPPLEMENTARY METHODS

Cells and cell culture

Melanoma cell lines were grown as reported in [1]. A375 C2, A375 P2, 501 Mel P1 and SK-Mel-28 C1 vemurafenib-resistant cells were cultured in presence of vemurafenib as reported in [2]. Vemurafenib (PLX-4032, 1029872-54-5) was purchased from Selleckchem (Selleckchem, Houston, Texas, USA). The identity of each cell line is confirmed by fingerprinting, as reported in [1] and [2].

Detection of LLO by western blot

To detect intracellular LLO, an overnight growth of Lm^{at}-LLO and Lm(ct) were pelleted, then resuspended in 500ul of water and 500ul of 2x NuPAGE® LDS Sample Buffer (NP0008, Thermo Fisher Scientific, Waltham, Massachusetts, USA). 30ul of proteins were boiled and loaded on 10% agarose gel (Bio-Rad, Hercules, California, USA). They were then transferred to a nitrocellulose membrane (Bio-Rad, Hercules, California, USA) using Trans Blot® Turbo (Bio-Rad, Hercules, California, USA). Endogenous (58KDa) and exogenous (48 KDa) LLO were detected using a mouse polyclonal anti-LLO antibody that recognizes full length LLO protein and has been developed in the Gravekamp lab. The secondary goat anti-mouse IgG HRP (sc-2005) was purchased from Santa Cruz (Santa Cruz, Santa Cruz, California, USA) and diluted according to the manufacturer's instructions.

Transmission electron microscope analysis

To visualize the life cycle of Lm^{at}-LLO, 501 Mel cells were seeded at $5x10^5$ cells/P100. The day after the medium was replaced with fresh DMEM supplemented with 10% foetal bovine serum, 1% glutamine without Penicillin/Streptomycin and the cells were infected with 200 MOI of Lm^{at}-LLO. After 2h, cells were treated with 50ug/ml of gentamycine and, after additional 6h, they were harvested and pelleted by centrifugation. Pellets were washed three times with PBS and fixed in 3%

glutaraldehyde solution in 0.1M cacodylate buffer, pH 7.2, for 2h at 4 °C. Cells were then scraped off and post-fixed in 1% osmium tetroxide in 0.1M cacodylate buffer for 2h at room temperature. After rapid dehydration in a graded series of Ethanol and propylene oxide, cells were embedded in an “Epon-Araldite” mixture. Ultrathin sections, obtained by a diamond knife on an Ultracut Reichert-Jung ultramicrotome, were placed on Formvar-carbon coated nickel grids, stained with uranyl acetate and lead citrate and observed with a Jeol 100 SX transmission electron microscope.

Immunofluorescence

$2x10^4$ 501 Mel cells were seeded in 8 well glass slide (Lab tek II Chamber slide system, Thermo Fisher Scientific, Waltham, Massachusetts, USA). The day after the medium was replaced with fresh DMEM supplemented with 10% foetal bovine serum, 1% glutamine (Sigma-Aldrich, Saint Louis, Missouri, USA) without Penicillin/Streptomycin and the cells were infected with MOI 3000 of Lm^{at}-LLO. After 2h, cells were treated with 50ug/ml of gentamycine (Sigma-Aldrich, Saint Louis, Missouri, USA) to kill the listeria that was not inside melanoma cells and were cultured for additional 1 or 4h. Then, they were fixed in 3.7% formaldehyde and permeabilized with 0.1% Triton X-100 in PBS. After the blocking (1% goat serum) and washing steps, they were incubated with primary anti-Listeria antibody (BD Difco Listeria antiserum poly serotypes 1 and 4, 223021, BD, Franklin Lakes, New Jersey, USA) for 90min at room temperature. The primary antibody was diluted in PBS supplemented with 0.03% BSA and 0.1% Triton X-100. After the washing steps, the incubation with the secondary antibody (goat anti-rabbit 594 IgG Alexa Fluor, Thermo Fisher Scientific, Waltham, Massachusetts, USA) was performed at room temperature for 60min. Cells were finally incubated with Alexa Fluor 488 Phallotoxin (40U/mL, Life Technologies, Carlsbad, California, USA) for 20min at RT to stain the cytoplasmic F-actin and with DAPI to stain nuclei. Slides were visualized on a Leica

SP2 confocal microscope equipped with an AOBS system using a 63× oil immersion objective. They were subsequently analyzed using ImageJ software.

In vitro infection rate

$5x10^5$ 501 Mel cells were seeded in 24well plates in DMEM supplemented with 10% foetal bovine serum, 1% glutamine without Penicillin/Streptomycin. After 4h, 200 MOI of Lm^{at}-LLO or Lm(ct) were added to the cells. Gentamycine (50ug/ml) was added after 2h of infection and cells were cultured for additional 1 or 4h. Cells were then washed three times with DMEM supplemented with 10% foetal bovine serum, 1% glutamine without Penicillin/Streptomycin. 1ml of sterile water was added to lysate tumor cells, then 50ul of undiluted and 1:10 diluted suspensions were plated on LB agar and listeria colonies were counted the next day.

Cell to cell spreading

$5x10^5$ of 501 Mel cells were seeded in 6well plates in DMEM supplemented with 10% foetal bovine serum, 1% glutamine without Penicillin/Streptomycin. After 24h, MOI 200 of Lm^{at}-LLO or Lm(ct) were added to the cells. Gentamycine (50 ug/ml) was added after 2h of infection and cells were cultured for additional 1 or 4h. Then, they were harvested and incubated with anti-Listeria antibody (BD Difco Listeria antiserum poly serotypes 1and 4, 223021, BD, Franklin Lakes, New Jersey, USA), followed by secondary antibody (goat anti-rabbit 594 IgG Alexa Fluor, Thermo Fisher Scientific, Waltham, Massachusetts, USA). Finally, fluorescence was measured by flow cytometry (CyFlow® Cube 8 flow cytometer, Sysmex Partec GmbH, Germany). The analysis of flow cytometry data was carried out with FCS-Express 4 software (De Novo Software).

Intracellular ROS production

$3.5x10^5$ 501 Mel cells were seeded in 6well plates. The day after they were infected with MOI 200 of Lm^{at}-LLO or Lm(ct) for 2h. Cells were then treated with 50ug/ml gentamycine and were cultured for 4 additional hours. Finally, CellRoX® Oxidative Stress Reagent (7uM, C10444, Life Technologies, Carlsbad, California, USA) was added, following the manufacturer's instructions. The day after, fluorescence was measured by flow cytometry (CyFlow® Cube 8 flow cytometer, Sysmex-Partec). The analysis of flow cytometry data was carried out with FCS-Express 4 software (De Novo Software, Glendale, California, USA).

AnnexinV/PI staining

$2x10^5$ 501 Mel cells were seeded in 6well plates. The day after, they were infected with MOI 100 or 200 of Lm^{at}-LLO for 2h. Cells were then treated with 50ug/ml gentamycine. The day after, cells were stained with 5ul of Annexin V–FITC and 3ul of Propidium Iodide (PI, 1mg/ml) for 30min at room temperature, according to the manufacturer's instructions (Sigma-Aldrich, Saint Louis, Missouri, USA). Then, fluorescence was measured by flow cytometry (CyFlow® Cube 8 flow cytometer, Sysmex Partec GmbH, Germany). The analysis of flow cytometry data was carried out with FCS-Express 4 software (De Novo Software, Glendale, California, USA).

Kill rate

Kill rate of melanoma cells after listeria infection was assessed plating $3x10^3$ melanoma cells in 96well plates. After 3h, MOI 300 and 3000 of Lm^{at}-LLO or Lm(ct) were added. Gentamycine (50ug/ml) was added 2h later. The day after, 10ul of trypan blue were added to each well and they were incubated for 10min at 37°C 5%CO_2. Alive (trypan blue negative) and dead cells (trypan blue positive) were counted in three different fields in each well. Each experiment was performed in triplicate, three times.

Detection of stem cell markers by flow cytometry

Melanoma cells were stained with: Rh123 (R8004, Sigma-Aldrich, Saint Louis, Missouri, USA), Aldefluor^{TM} assay (01700, Stemcell Technologies, Vancouver, Canada), anti-CD166-VioBright FITC (clone REA442, 130-106-621, Miltenyi Biotec, Bergisch Gladbach, Germany), anti-CD271 (LNGFR)-PE-Vio770 (clone REA648, 130-110-113, Miltenyi Biotec, Bergisch Gladbach, Germany). For each sample, 100.000 events were analysed by flow cytometry using BD FACS JAZZ (BD, Franklin Lakes, New Jersey, USA). Analysis of flow cytometry data was carried out with BD FACS JAZZ software (BD, Franklin Lakes, New Jersey, USA).

Stable infection

The stable infection of pGIPZ-tGFP and pGIPZ-mCherry lentiviral vectors (kind gifts from Dr. Hernando, NYU) was carried out as described in [3].

Cell sorting

10^7 SK-Mel-5-pGIPZ-mCherry cells, as well as 10^7 SK-Mel-2-pGIPZ-tGFP or SK-Mel-28-pGIPZ-tGFP cells were stained with anti-CD166-VioBright FITC (clone REA442, 130-106-621, Miltenyi Biotec, Bergisch Gladbach, Germany) and anti-CD271 (LNGFR)-PE-Vio770 (clone REA648, 130-110-113, Miltenyi Biotec, Bergisch Gladbach, Germany) respectively, according to the manufacturer's instructions. Labelled cells were then sorted with BD FACS JAZZ (BD, Franklin Lakes, New Jersey, USA) in two populations (CD166/CD271 positive and negative populations), according to their fluorescence level. Cell aggregates and dead cells were excluded by gating FSC and SSC. Analysis of sorting data was carried out with BD FACS JAZZ software (BD, Franklin Lakes, New Jersey, USA). After sorting, cells were resuspended in cell medium, counted and used for appropriate experiments.

Clonogenicity assay

Right after sorting, $2x10^2$ SK-Mel-5 cells (CD166 pos. and CD166 neg.) were seeded in 60mm plates in triplicate. After 14 days, cells were fixed and stained with a 0.1% crystal violet, 4% formaldehyde solution.

Xenograft in zebrafish embryos

$2.5x10^5$ SK-Mel-2 and SK-Mel-28 melanoma cells sorted for CD271 (CD271 pos. and CD271 neg.) were resuspended in 2ul of matrigel (Cultrex Basement Membrane Extract, PathClear, Sigma-Aldrich, Saint Louis, Missouri, USA) and injected into the perivitelline space of 48hpf zebrafish embryos of the Tg(myil7:DsRed) strain as described in [2].

RNA extraction and quantification

RNA extraction from cell lines and liver of heathy mice: RNA was extracted using QIAzol reagent (Qiagen, Hilden, Germany), following the manufacturer's instructions.

RNA extraction from paraffin embedded samples: for each paraffin embedded specimen, 10 slices 20um thick were cut. 1ml of 100% xylene was added and the sample was incubated at 50°C for 3min, then centrifuged. This step was repeated three times. Pellets were washed twice with 1ml of 100% ethanol and air dried. Subsequently, they were incubated with 150ul of digestion buffer (20 mM Tris-HCl (pH8.0), 1mM $CaCl_2$, 0.5% sodium dodecyl sulfate, 500ng/ml Protease K (P4850, Sigma-Aldrich, Saint Louis, Missouri, USA)) at 55°C for 3h. At the end of the incubation period, 1ml of QIAzol (Qiagen, Hilden, Germany) was added and incubated at 30°C for 5min. After the addition of 200ul of chloroform, samples were vortexed for 15sec. They were then centrifuged at 12000xg for 15min at 4°C and the aqueous phase was transferred to a fresh tube adding 10ug of glycogen. Total RNA was precipitated by adding 600ul of isopropyl alcohol and incubating the sample at -20°C for at least 1h. It was then centrifuged at 12000xg for 10min at 2-8°C, washed with 100% ethanol and dissolved in 20ul of RNase-free water or TE.

RNA was subsequently quantified using Nanodrop Lite (Thermo Fisher Scientific, Waltham, Massachusetts, USA).

DNAse treatment and retrotranscription

1ug of RNA was treated with DNAse I, amplification grade (Invitrogen) following the manufacturer's instructions. 500ng of DNAse-treated RNA were then retrotranscribed with iScript cDNA Synthesis Kit (Bio-Rad, Hercules, California, USA) using a S1000 Thermal Cycler (Bio-Rad, Hercules, California, USA) [2].

Real-time PCR

qRT-PCR was performed with SsoAdvanced Universal Supermix (Bio-Rad, Hercules, California, USA) on a CFX96 Real-Time System (Bio-Rad, Hercules, California, USA). All reactions were performed in duplicate. Data were analyzed using CFX Manager Software (Bio-Rad, Hercules, California, USA), as described in [2]. The primers used are reported in **Supplementary Table 1**.

Genomic DNA extraction and genotyping of mice

A piece of tail collected from 4 weeks old mice was resuspended in 500ul of Lysis Buffer pH8.0 (10mM Tris-HCl, 400mM NaCl, 2mM EDTA) plus 40ul of 10% SDS and 20ul of 1mg/ml RNAse. Samples were incubated at 37°C for 1h and then incubated with 10ul of proteinase K (P4850, Sigma-Aldrich, Saint Louis, Missouri, USA) at 50°C for 1h. Saturated NaCl (200ul) were added and samples were then centrifuged at 12000rpm for 10min. Supernatant was collected and 2 volumes of 100% Ethanol were added. Samples were centrifuged for 5min at 12000rpm. The obtained pellet was then washed with 70% Ethanol and resuspended in 50-80ul of TE buffer. DNA was subsequently quantified using Nanodrop Lite (Thermo Fisher Scientific, Waltham, Massachusetts, USA). The PCR for genotyping were performed as described in https://www.jax.org/strain/013590, using Phusion High-Fidelity DNA

Polymerase (Thermo Fisher Scientific, Waltham, Massachusetts, USA) and the primers listed in **Supplementary Table 1**.

LD50 calculation

The Lethal Dose 50 (LD50) of Lm^{at}-LLO was determined by vaccinating 20 mice with 10^7, 10^8 and $5x10^8$ CFU of Lm^{at}-LLO in 100ul of saline solution (0.9% NaCl) (4 experiments, n=5 mice for each dose). Mice were monitored for three days. After that, they were suppressed and their liver was analysed by visual inspection, as well as collected for Hematoxylin/Eosin staining.

Ultrasound imaging

Ultrasound imaging was performed using a Vevo 2100 ultrasound system (FUJIFILM VisualSonics Inc., Toronto, Canada) under isoflurane inhalation anesthesia (induction: 2.5%, maintenance: 1.5%; flow 1 l/min). Animals were previously shaved using depilatory cream and fixed on a heated stand. Temperature and both respiration and heart rate were monitored during the acquisition (THM-100; Indus Instruments, Houston, USA). MS550 probe (22-55 MHz) was coupled to mice with acoustic gel and it was used for all acquisitions. B-mode and Power Doppler-mode images from primary tumor, inguinal lymph nodes and skin metastasis (if present) were acquired in short axis view positioning the animals in dorsal/ventral recumbency according to the scanned target. Power Doppler was used to qualitatively assess the vascularity. Furthermore, 3D-mode acquisitions of the inguinal lymph nodes (at regular spatial 44µm intervals) were obtained. 3D scans were imported in VevoLab software platform (FUJIFILM VisualSonics Inc., Toronto, Canada) and lymph nodes were identified and marked in the acquired cross-sectional 2D images semi-automatically. Finally, lymph node volumes were calculated by the volumetric analysis function of the VevoLab software from multiple marked tumor perimeters.

Histology and immunohistochemistry

Tissues were collected and fixed in 4% formaline for at least 48h. They were then kept in 70% Ethanol overnight at 4°C and in 95% Ethanol for 4h. Fresh 95% Ethanol was substituted and tissues were stored overnight at room temperature. After that, 99% Ethanol was used for 1h at room temperature. Finally, samples were incubated in xylene at room temperature, first for 2-3h and then overnight. The day after, the tissues were included in paraffin. Sections of 5um thickness were stained with Hematoxylin (05-M06002, Bio-Optica Milano Spa, Milan, Italy) and Eosin (05-B10003, Bio-Optica Milano Spa, Milan, Italy). Single immunostainings were performed on adjacent serial sections by using immunoperoxidase technique, 3-3′ diaminobenzidine chromogen substrate, Haematoxylin counterstaining and specific antibodies raised against MLANA (M2-7C10, Thermo Fisher Scientific, Waltham, Massachusetts, USA), BRAFV600E (E19290, Spring Bioscience Corporation, Pleasanton, California, USA), CD8 (MA1-10301, Thermo Fisher Scientific, Waltham, Massachusetts, USA), CD3 (ab5690, Abcam, Cambridge, UK), CD4 (4SM95, Life Technologies, Carlsbad, California, USA), Ki-67 (ab15580, Abcam, Cambridge, UK) or Cleaved Caspase-3 (D175, Cell Signaling Technology Inc., Danvers, Massachusetts, USA) and examined under light microscope (Olympus BX43). Melanin was removed by incubating slices with 1% KOH and 3% H_2O_2 for 1h at room temperature. For MLANA and BRAFV600E immunostainings, antigen retrival was performed by microwave heating in NaCitrate pH6.0 (Thermo Fisher Scientific, Waltham, Massachusetts, USA) and EDTA Buffer pH8.0, respectively. Ki-67 and Cleaved Caspase-3 staining were quantified as ratio between positive cells and total cell number.

***In vivo* infection rate**

About 2x3x2mm of tissues of Lm^{at}-LLO injected mice (those indicated in **Supplementary Fig. 13a and 18**) were collected and washed in PBS. About 0.01g of

each tissue was then incubated with 100ul of 10X trypsin for 20 min at 37°C. After being centrifuged to remove the trypsin, it was mechanically dissociated and resuspended in 200ul of water. After 5min, the suspension was seeded on LB agar plates with 20ug/ml chloramphenicol. Blood (100ul) was seeded on LB agar plates with 20ug/ml chloramphenicol. Colonies were counted after 24h.

REFERENCES

1 Marranci A, Tuccoli A, Vitiello M, Mercoledi E, Sarti S, Lubrano S *et al*. Identification of BRAF 3'UTR Isoforms in Melanoma. J Invest Dermatol 2015; 135: 1694-1697.

2 Vitiello M, Tuccoli A, D'Aurizio R, Sarti S, Giannecchini L, Lubrano S *et al*. Context-dependent miR-204 and miR-211 affect the biological properties of amelanotic and melanotic melanoma cells. Oncotarget 2017; 8: 25395-25417.

3 Gaziel-Sovran A, Segura MF, Di Micco R, Collins MK, Hanniford D, Vega-Saenz de Miera E *et al*. miR-30b/30d regulation of GalNAc transferases enhances invasion and immunosuppression during metastasis. Cancer Cell 2011; 20: 104-118.
