# Supplementary material for: Antitumoral effects of attenuated Listeria monocytogenes in a genetically engineered mouse model of melanoma
Source: Oncogene. 2019 Jan 21;38(19):3756–62. doi: 10.1038/s41388-019-0681-1 (PMC6756113; doi:10.1038/s41388-019-0681-1)
Supplement: Supplementary file 3 — Supplementary Table S1 [file 41388_2019_681_MOESM3_ESM.pdf]

Supplementary Table 1. List of primers used for mutational analysis of melanoma cell lines (I), mice genotyping (II) and qRT-PCR (III).

I

Mutated gene	Residue	Exon	Forward primer	Reverse primer
hsaBRAF	L505	12	CATGGAACAAACAAGGTTGG	AGTTGCTACCACTGGGAACC
	V600	15	TCATAATGCTTGCTCTGATAGGA	GGCCAAAAATTTAATCAGTGGA
hsaNRAS	G12	2	CTGGTTTCCAACAGGTTCTTGC	CTACCACTGGGCCTCACCT
	Q61	3	CATACTGGATACAGCTGGAC	TGACTTGCTATTATTGATGG
	A146	4	TGCCCAGGCTAATCTCAAAC	TCACTTGAACCCAAGAGACAGA

II

Gene	Forward primer	Reverse primer
Braf	TGAGTATTTTTGTGGCAACTGC	CTCTGCTGGGAAAGCGGC
Pten	CAAGCACTCTGCGAACTGAG	AAGTTTTTGAAGGCAAGATGC
CRE	GCGGTCTGGCAGTAAAAACTATC	GTGAAACAGCATTGCTGTCACTT
Internal Positive Control	CTAGGCCACAGAATTGAAAGATCT	GTAGGTGGAAATTCTAGCATCATCC

III

Gene	qRT-PCR forward primer	qRT-PCR reverse primer
hsaNF1	CCATGGAATTGTGCAGAGTG	TTGAAAACGGTCCTGCAAAC
	CTGAGCACAACAAGGAATGTC	TTTCAGCAGCTTCTCCAAAT
hsaABCB5	GCTGAGGAATCCACCCAATCT	CACAAAAGGCCATTCAGGCT
hsaALDH1A1	GCATCCAGGATTTTTGTGGA	TCCCACTCTCAATGAGGTCAA
hsaALDH1A3	GCATGAGCCCATTGGTGTCT	CGCAGGCTTCAGGACCAT
hsaCD133	CTGGGGCTGCTGTTTATTA	TACCTGGTGATTTGCCACAA
hsaCD166	TGATCTCCGCCACCGTCTTCAG	CTCTTTTCATCACTGATCCTTGCA
hsaCD271	CTCATCCCTGTCTATTGCTC	CTCCTTGCTTGTTCTGCTT
hsaGAPDH	*CGCTCTCTGCTCCTCCTGTT*	*CCATGGTGTCTGAGCGATGT*
hsaPBGD	*TCCAAGCGGAGCCATGTCTG*	*AGAATCTTGTCCCCTGTGGTGGA*
hsaSDHA	*CCACTCGCTATTGCACACC*	*CACTCCCCATTCTCCATCA*
mmuMLANA	TTATCGGCTGCTGGTACTGT	CGGGCTGATGGGATTTCTCT
mmuGP100	GGCACACACACAATGGAAGT	AGGAAGTGCTTGGTCTCTCC
mmuS100	TGTCTTCCACCAGTACTCCG	TCCAGCGTCTCCATCACTTT
mmuPten-ex3/4-5	TGGATTCAAAGCATAAAAACCATTAC	CAAAAGGATACTGTGCAACTCTGC
mmuIL-2	AGCAGCTGTTGATGGACCTA	CGCAGAGGTCCAAGTTCAT
mmuIL-8	GCGCCCAGACAGAAGTCATAG	GGCAAACTTTTTGACCGCC
mmup56	TCAAGTATGGCAAGGCTGTG	GAGGCTCTGCTTCTGCATCT
mmuHPRT	*GGAGCGGTAGCACCTCCT*	*AACCTGGTTCATCATCGCTAA*
mmuAct	*CTAAGGCCAACCGTGAAAAG*	*ACCAGAGGCATACAGGGACA*
mmuGAPDH	*GCCTTCCGTGTTCCTACCC*	*TGCCTGCTTCACCACCTC*
